# Longitudinal MRI‐Driven Multi‐Modality Approach for Predicting Pathological Complete Response and B Cell Infiltration in Breast Cancer

**DOI:** 10.1002/advs.202413702

**Published:** 2025-02-07

**Authors:** Yu‐Hong Huang, Zhen‐Yi Shi, Teng Zhu, Tian‐Han Zhou, Yi Li, Wei Li, Han Qiu, Si‐Qi Wang, Li‐Fang He, Zhi‐Yong Wu, Ying Lin, Qian Wang, Wen‐Chao Gu, Chang‐Cong Gu, Xin‐Yang Song, Yang Zhou, Dao‐Gang Guan, Kun Wang

**Affiliations:** ^1^ Department of Breast Cancer Cancer Center Guangdong Provincial People's Hospital (Guangdong Academy of Medical Sciences) Southern Medical University No. 106 Zhongshan Second Road, Yuexiu District Guangzhou Guangdong Province 510080 China; ^2^ Department of Biochemistry and Molecular Biology School of Basic Medical Sciences Southern Medical University Guangzhou Guangdong Province 510515 China; ^3^ Guangdong Key Laboratory of Single Cell Technology and Application Southern Medical University, Guangzhou Guangdong Province 510515 China; ^4^ The Department of General Surgery Hangzhou TCM Hospital Affiliated to Zhejiang Chinese Medical University Xihu District Hangzhou Zhejiang Province 310000 China; ^5^ Galactophore Department Jingzhou Hospital Affiliated to Yangtze University Shashi District Jingzhou 434000 China; ^6^ Department of Biostatistics Harvard T.H. Chan School of Public Health Boston MA 02115 USA; ^7^ Breast Center Cancer Hospital of Shantou University Medical College Jinping District Shantou Guangdong Province 515000 China; ^8^ Clinical research center & Breast disease diagnosis and treatment center Shantou Central Hospital No. 114 Waima Road, Jinping District Shantou Guangdong Province 515000 China; ^9^ Breast Disease Center, The First Affiliated Hospital Sun Yat‐sen University No. 58 Zhongshan Second Road, Yuexiu District Guangzhou Guangdong Province 510080 China; ^10^ Department of Radiology The Affiliated Huaian No.1 People's Hospital of Nanjing Medical University Huaiyin District Huaian Jiangsu Province 223001 China; ^11^ Department of Artificial Intelligence Medicine Graduate School of Medicine Chiba University Chiba 263‐8522 Japan; ^12^ Department of Medical Imaging The First Hospital of Qinhuangdao Haigang District Qinhuangdao Hebei Province 066000 China; ^13^ Department of Radiology The First Affiliated Hospital of Jinan University No. 613 Huangpu West Road, Tianhe District Guangzhou Guangdong 510627 China; ^14^ Department of Pathology The Second People's Hospital of Changzhou, The Third Affiliated Hospital of Nanjing Medical University No. 29 Xinglong Lane Changzhou Jiangsu Province 213164 China

**Keywords:** artificial intelligence, breast cancer, medical imaging, multi‐omics analysis, neoadjuvant treatment

## Abstract

Accurately predicting pathological complete response (pCR) to neoadjuvant treatment (NAT) in breast cancer remains challenging due to tumor heterogeneity. This study enrolled 2279 patients across 12 centers and develops a novel multi‐modality model integrating longitudinal magnetic resonance imaging (MRI) spatial habitat radiomics, transcriptomics, and single‐cell RNA sequencing for predicting pCR. By analyzing tumor subregions on multi‐timepoint MRI, the model captures dynamic intra‐tumoral heterogeneity during NAT. It shows superior performance over traditional radiomics, with areas under the curve of 0.863, 0.813, and 0.888 in the external validation, immunotherapy, and multi‐omics cohorts, respectively. Subgroup analysis shows its robustness across varying molecular subtypes and clinical stages. Transcriptomic and single‐cell RNA sequencing analysis reveals that high model scores correlate with increased immune activity, notably elevated B cell infiltration, indicating the biological basis of the imaging model. The integration of imaging and molecular data demonstrates promise in spatial habitat radiomics to monitor dynamic changes in tumor heterogeneity during NAT. In clinical practice, this study provides a noninvasive tool to accurately predict pCR, with the potential to guide treatment planning and improve breast‐conserving surgery rates. Despite promising results, the model requires prospective validation to confirm its utility across diverse patient populations and clinical settings.

## Graphical abstract

This study develops a multi‐modality model integrating longitudinal magnetic resonance imaging, transcriptomic, and scRNA‐seq data to predict pathological complete response (pCR) in breast cancer. It outperforms traditional radiomics models, offering non‐invasive pCR prediction and help guide treatment decisions. The scRNA‐seq analysis reveals the biological basis of the model, specifically indicating that higher model scores are associated with increased B cell infiltration.

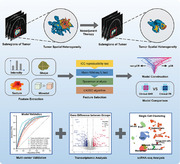

## 1 Introduction

Breast cancer is the most common malignancy among women, and its incidence continues to rise worldwide.^[^ ^1^ ^]^ Neoadjuvant treatment (NAT) has become the first‐line treatment for patients with early high‐risk and locally advanced breast cancer.^[^ ^2^ ^]^ Achieving a pathological complete response (pCR) is a critical indicator for successful NAT, as it not only facilitates breast‐conserving surgery but also improves event‐free and overall survival rates.^[^ ^3^, ^4^ ^]^ However, due to tumor heterogeneity, the response to NAT varies widely among patients, with reported pCR rates ranging from 35% to 60%.^[^ ^5^ ^]^ Therefore, it is important to accurately predict the tumor response to NAT, particularly for patients at risk of undergoing prolonged chemotherapy and experiencing associated adverse effects.

Multiple clinical and pathological factors have been explored to predict pCR, including tumor size, grade, and receptor status, but these factors often lack precision. Breast magnetic resonance imaging (MRI) is the preferred method for evaluating tumor response during NAT, as it offers a more comprehensive view of tumor extent and morphology than mammography or ultrasound.^[^ ^6^ ^]^ Dynamic contrast‐enhanced MRI is widely used because it effectively distinguishes tumor from normal tissue based on hemodynamic and morphological characteristics. Functional imaging techniques, such as diffusion‐weighted imaging (DWI) and magnetic resonance spectroscopy, have broadened MRI's role in diagnosing and monitoring breast cancer.^[^ ^7^, ^8^, ^9^ ^]^ A study reported that DWI achieved higher diagnostic performance than standard mammography in breast cancer diagnosis,^[^ ^10^ ^]^ while time‐dependent diffusion MRI‐based microstructural mapping accurately predicted both molecular subtypes and pCR.^[^ ^11^ ^]^ Moreover, 18F‐FDG PET/MRI has shown greater sensitivity than MRI alone for monitoring tumor response.^[^ ^12^ ^]^ In recent years, radiomics has been widely applied to extract quantitative features from medical images for cancer diagnosis, tumor response monitoring, and prognosis prediction.^[^ ^13^, ^14^, ^15^, ^16^ ^]^ By quantifying tumor heterogeneity and revealing potential imaging patterns not visible to the naked eye, radiomics can improve diagnostic precision. However, traditional whole‐tumor radiomics analysis cannot fully quantify tumor heterogeneity, as it overlooks subregional differences within the tumor. Thus, more advanced approaches that capture intratumoral heterogeneity are urgently needed to improve prediction accuracy.

Spatial habitat radiomics (SHR) divides tumors into distinct habitats based on imaging characteristics, reflecting differences in cell density, metabolic activity, and blood perfusion.^[^ ^17^, ^18^, ^19^ ^]^ Integrating spatial habitat analysis has been shown to increase the accuracy of radiomics models, offering more detailed insights into tumor biology.^[^ ^20^, ^21^ ^]^ However, most current studies rely on single time‐point imaging and overlook dynamic tumor changes during treatment. Because tumor heterogeneity evolves over time, traditional radiomics fails to fully capture temporal changes relevant to treatment. In contrast, multiple time‐point imaging analyses could better capture this temporal heterogeneity and develop more precise models.^[^ ^13^, ^22^, ^23^, ^24^ ^]^ Additionally, correlating imaging features with gene expression, protein profiles, and single‐cell sequencing helps reveal the molecular mechanism driving the imaging phenotypes,^[^ ^25^, ^26^, ^27^ ^]^ potentially identifying novel therapeutic targets. Single‐cell RNA sequencing (scRNA‐seq) further clarifies intra‐tumoral heterogeneity and deepens our understanding of the tumor microenvironment. However, no study has integrated single‐cell data with multiple time‐point imaging data to explore noninvasive imaging biomarkers for predicting tumor response.

This study aims to develop a noninvasive multi‐modality model using MRI scans at multiple time‐points to predict pCR in breast cancer. We compared the spatial habitat radiomics model with traditional radiomics to assess its clinical value for identifying pCR patients. Furthermore, we integrated transcriptomics and scRNA‐seq data to explore the biological basis of the model. This combined analysis deepened our understanding of the tumor immune microenvironment, especially how imaging features reflect immune cell infiltration and tumor response to NAT.

## 2 Results

### 2.1 Patient Characteristics

**Table** **1** presents the patient characteristics. A total of 2279 patients were included: 431 in the training cohort (median age, 48 years [IQR, 42‐56]) and 1848 in the validation cohort (median age, 50 years [IQR, 42‐57]). The median tumor sizes on pre‐NAT and mid‐NAT MRI were 5.1 cm (range, 0.9–13.2 cm) and 3.0 cm (range, 0–12.5 cm), respectively. Pathological assessments showed that 904 patients (39.6%) achieved pCR. The cT1, cN0, high Ki‐67 expression, ER negativity, and HER2 positivity were all significantly associated with pCR (*p* <0.05), while age, menstrual status, and tumor grade did not differ between the pCR and non‐pCR groups. Baseline characteristics were generally similar in both cohorts, except for molecular subtypes (*p* <0.05). Overall, the diversity of the population helped evaluate the model more comprehensively.

**Table 1:** Comparison of Patient Characteristics among Different Cohorts.

Characteristics	Training Cohort	Validation Cohort	Immunotherapy Cohort	Multi‐omics Cohort	*p*‐value
Age at diagnosis (y)^a)^	48 (42, 56)	50 (42, 57)	49 (41, 56)	51 (43, 57)	0.162^d)^
Interval between MRIs (d)^b)^	83 (66, 103)	87 (70, 104)	84 (70, 95)	89 (72, 104)	0.575 **^d^** **^)^**
Pre‐NAT tumor size (cm)^b)^	5.1 (1.2, 11.3)	5.3 (1.2, 13.2)	4.9 (0.9, 10.5)	5.1 (1.1, 11.7)	0.329^d)^
Mid‐NAT tumor size (cm)^b)^	2.9 (0, 10.4)	3.3 (0, 12.5)	2.2 (0, 10.0)	2.4 (0, 10.8)	**0.006** ^d)^
Menopausal status					**< 0.001** ^c)^
premenopausal	231 (53.6)	999 (62.6)	56 (63.6)	78 (47.3)	
postmenopausal	200 (46.4)	596 (37.4)	32 (36.4)	87 (52.7)	
Clinical T stage					**< 0.001** ^c)^
cT1	29 (6.7)	103 (6.5)	9 (10.2)	10 (6.1)	
cT2	319 (74.0)	960 (60.2)	53 (60.2)	122 (73.9)	
cT3	65 (15.1)	340 (21.3)	18 (20.5)	28 (17.0)	
cT4	18 (4.2)	192 (12.0)	8 (9.1)	5 (3.0)	
Clinical N stage					**< 0.001** ^c)^
cN0	174 (40.4)	284 (17.8)	33 (37.5)	70 (42.4)	
cN1	211 (49.0)	828 (51.9)	32 (36.4)	76 (46.1)	
cN2‐3	46 (10.7%)	483 (30.3)	23 (26.2)	19 (11.5)	
Histological grade					**< 0.001** ^c)^
low (grade I)	12 (2.8)	17 (1.1)	0 (0)	0 (0)	
intermediate (grade II)	242 (56.2)	995 (62.4)	23 (26.1)	89 (53.9)	
high (grade III)	177 (41.1)	583 (36.6)	65 (73.9)	76 (46.1)	
ER status					**< 0.001** ^c)^
positive	290 (67.3)	927 (58.1)	0 (0)	63 (38.2)	
negative	141 (32.7)	668 (41.9)	88 (100)	102 (61.8)	
PR status					**< 0.001** ^c)^
positive	246 (57.1)	814 ^[^ ^51^ ^]^	0 (0)	45 (27.3)	
negative	185 (42.9)	781 ^[^ ^49^ ^]^	88 (100)	120 (72.7)	
HER2 status					**< 0.001** ^c)^
positive	161 (37.4)	775 (48.6)	0 (0)	98 (59.4)	
negative	270 (62.6)	820 (51.4)	88 (100)	67 (40.6)	
Ki‐67 index					0.645
high (≥20%)	385 (89.3)	1417 (88.8)	82 (93.2)	146 (88.5)	
low (<20%)	46 (10.7)	178 (11.2)	6 (6.8)	19 (11.5)	
Molecular subtype					**< 0.001** ^c)^
HER2+	161 (37.4)	775 (48.6)	0 (0)	98 (59.4)	
HR+/HER2‐	211 (49.0)	526 (33.0)	0 (0)	0 (0)	
TN	59 (13.6)	294 (18.4)	88 (100)	67 (40.6)	
Pathological response					**< 0.001** ^c)^
pCR	167 (38.7)	590 (37.0)	55 (62.5)	92 (55.8)	
non‐pCR	264 (61.3)	1005 (63.0)	33 (37.5)	73 (44.2)	

^a)^ The age was described as medians with interquartile ranges;

^b)^ The interval between MRI scans and tumor maximum diameter was described as means with ranges;

^c)^ p‐value was calculated using Fisher's exact test or Pearson's χ^2^ test comparing training cohort and integrated validation cohorts;

^d)^ *p*‐value was calculated using Kruskal‐Wallis test comparing training cohort and integrated validation cohorts. Abbreviation: NAT = neoadjuvant treatment, ER = estrogen receptor, PR = progesterone receptor, HR = hormone receptor, HER2 = human epidermal growth factor receptor 2, pCR = pathological complete response, MRI = magnetic resonance imaging.

**Figure 1:**
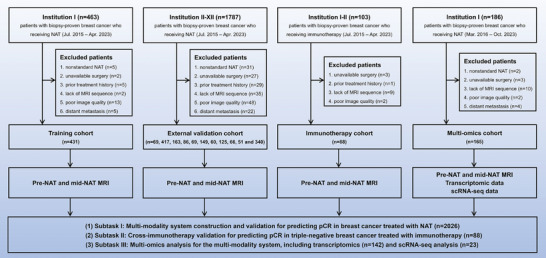
The flowchart of patient inclusion and exclusion in this study. Four cohorts: training (*n* = 431), external validation (*n* = 1595), immunotherapy (*n* = 88), and multiomics cohort (*n* = 165) are used for artificial intelligence model construction, independent validation, and biological interpretability analysis. NAT = neoadjuvant treatment, pCR = pathological complete response, MRI = magnetic resonance imaging.

**Figure 2:**
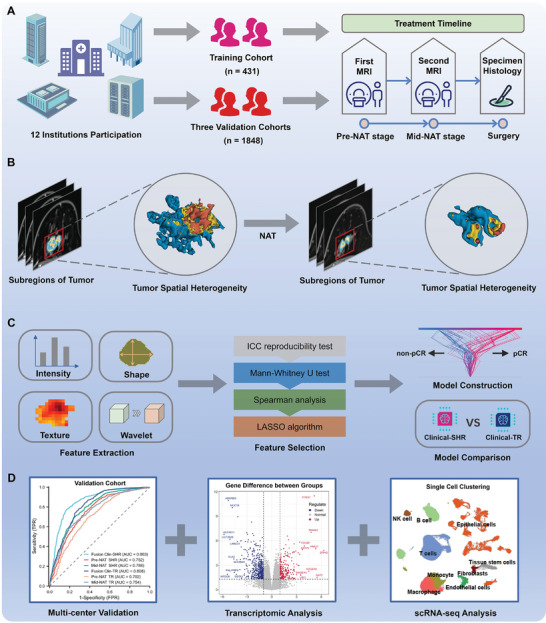
Overview of the study design and workflow. A) Breast cancer patients from 12 institutions were divided into a training cohort (*n* = 431) and three validation cohorts (*n* = 1825). Pre‐NAT and mid‐NAT MRI scans were obtained, followed by histological analysis after surgery. B) Tumor subregions were analyzed for spatial heterogeneity before and after NAT. C) Feature extraction included intensity, shape, texture, and wavelet features, followed by ICC reproducibility tests, Mann‐Whitney U tests, Spearman analysis, and LASSO for feature selection. Models were constructed and compared. D) Clinical validation, transcriptomic, and scRNA‐seq analysis were performed to evaluate the model's biological relevance. NAT = neoadjuvant treatment, pCR = pathological complete response, MRI = magnetic resonance imaging, ICC = intraclass correlation coefficient, LASSO = Least Absolute Shrinkage and Selection Operator.

The study flowchart is shown in **Figure** **1**. Center I (*n* = 431) provided the training cohort, while centers II‐XII (*n* = 69, 417, 163, 86, 69, 149, 60, 125, 66, 51, and 340, respectively) provided the external validation cohort. In addition, 88 patients from centers I and II who received neoadjuvant immunotherapy were enrolled as the immunotherapy cohort. Another 165 patients from Center I were included in the multi‐omics cohort, comprising 142 with transcriptome data and 23 with single‐cell sequencing data. The study workflow is depicted in **Figure** **2**.

### 2.2 Feature Selection

We extracted quantitative imaging features from the whole tumor (traditional radiomics [TR]) and from each tumor subregion (spatial habitat radiomics [SHR]), resulting in a total of 9784 features: 1223 pre‐NAT TR, 3669 pre‐NAT SHR, 1223 mid‐NAT TR, and 3669 mid‐NAT SHR features. After inter‐reader and intra‐reader Intraclass Correlation Coefficient (ICC) analysis, 1139 pre‐NAT TR (average ICC: 0.872), 3420 pre‐NAT SHR (average ICC: 0.881), 1127 mid‐NAT TR (average ICC: 0.869), and 3377 mid‐NAT SHR (average ICC: 0.858) features were retained with ICC value >0.8. After the Mann–Whitney U test (*p* < 0.05) and Spearman correlation analysis (correlation coefficient > 0.9), 1860 features from the pre‐NAT MRI and 2102 features from the mid‐NAT MRI were retained. The least absolute shrinkage and selection operator (LASSO) regression then selected 8 pre‐NAT TR, 7 pre‐NAT SHR, 9 mid‐NAT TR, and 8 mid‐NAT SHR features for constructing single time‐point models (Figures S1 and S2, Supporting Information), and Spearman correlation analysis confirmed that all features were independent predictors of pCR (Figures S3 and S4, Supporting Information).

For the spatial habitat radiomics, we used k‐means clustering with the Calinski‐Harabasz index to divide each tumor into three subregions:^[^ ^1^ ^]^ High Metabolic Subregion (Region I), which indicates areas with high vascularity and metabolic activity; ^[^ ^2^ ^]^ Junction Subregion (Region II), representing transitional zones with moderate vascularity; and^[^ ^3^ ^]^ Marginal Subregion (Region III), indicating hypoxic and/or necrotic zones. We extracted texture, intensity, and shape features from each subregion as spatial habitat radiomics features. For instance, notable features included pre‐NAT GLCM inverse variance in Region I, which highlights uniformity in vascular areas, and mid‐NAT short‐run emphasis in Region II, which captures therapy‐related changes in perfusion and cellular composition.

### 2.3 Development of Single Time‐Point Models

**Figure 3:**
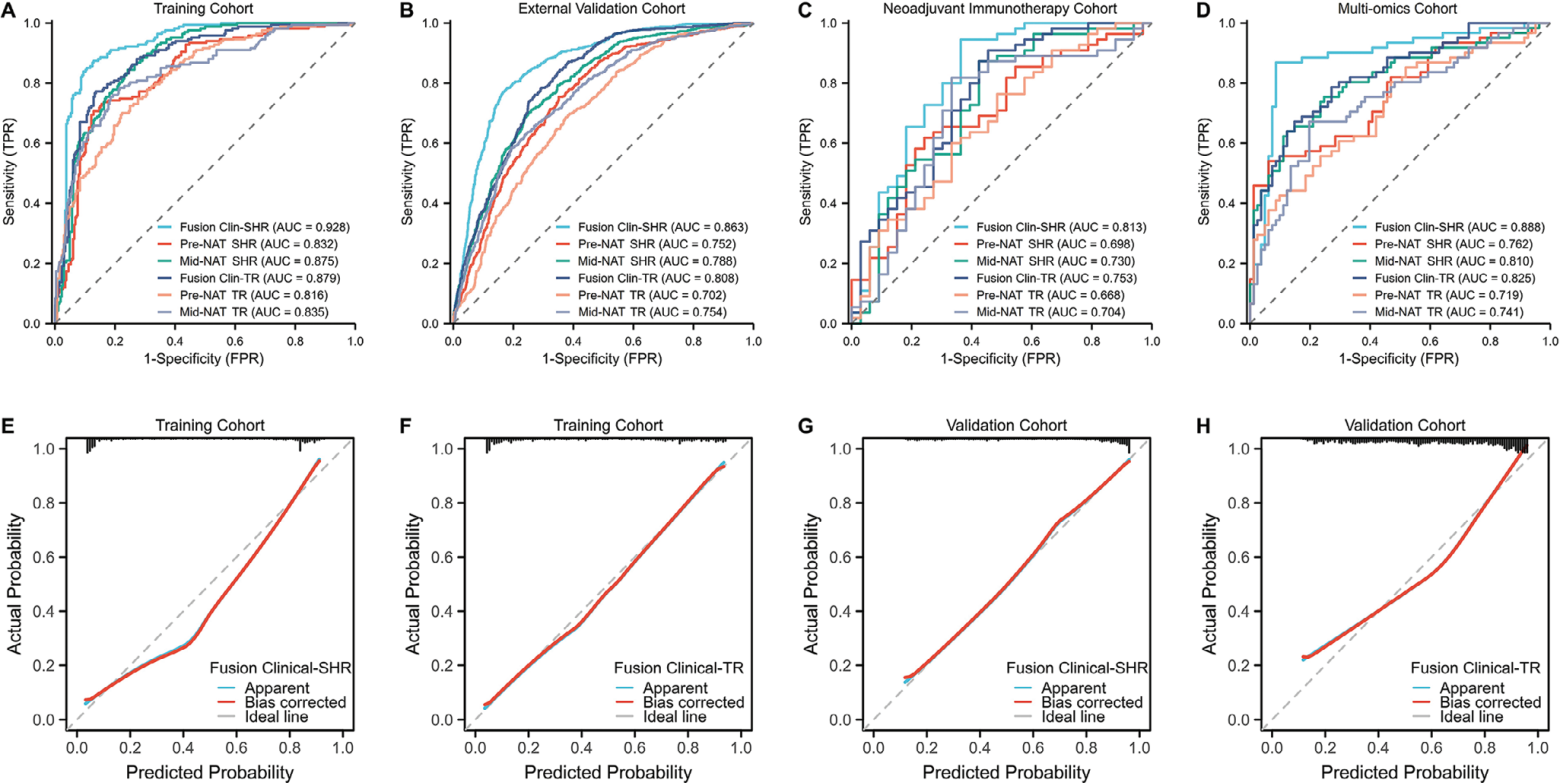
Model performance of artificial intelligence models in different cohorts. A–D) ROC curves for the training, external validation, neoadjuvant immunotherapy, and multi‐omics cohorts, respectively, showing the AUC for various models. E–H) Calibration curves for the training and validation cohorts comparing predicted probabilities and actual outcomes for Clin‐SHR and Clin‐TR models, demonstrating the models' predictive accuracy. NAT = neoadjuvant treatment, SHR = spatial habitat radiomics, TR = traditional radiomics, AUC = the area under the curve.

The four single time‐point models were:^[^ ^1^ ^]^ Pre‐NAT TR, using whole‐tumor radiomics features before NAT;^[^ ^2^ ^]^ Pre‐NAT SHR, using spatial habitat radiomics features before NAT;^[^ ^3^ ^]^ Mid‐NAT TR, using whole‐tumor radiomics features at the mid‐time of NAT; and^[^ ^4^ ^]^ Mid‐NAT SHR, using spatial habitat radiomics features at the mid‐timepoint of NAT. Those models showed distinct performances across different cohorts (**Table** **2**; Table S1, Supporting Information). In the training cohort, the pre‐NAT SHR and TR models had the area under the ROC curve (AUC) of 0.832 (95% CI: 0.792‐0.871) and 0.816 (95% CI: 0.776‐0.856), while the mid‐NAT SHR and TR models had the AUCs of 0.875 (95% CI: 0.843‐0.908) and 0.835 (95% CI: 0.795‐0.875). In the external validation cohort, the pre‐NAT SHR model achieved an AUC of 0.752 (95% CI: 0.728, 0.776), with a sensitivity of 75.42% (445/590) and a specificity of 64.88% (652/1105), outperforming the pre‐NAT TR model. The mid‐NAT SHR model obtained an AUC of 0.788 (95% CI: 0.766 to 0.811) with a sensitivity of 70.17% (414/590), surpassing the mid‐NAT TR model. In both the immunotherapy and multi‐omics cohorts, the SHR models also outperformed the TR models. **Figure** **3** shows the ROC curves and calibration curves of models in different cohorts.

**Table 2:** Comparison of Models’ Performance among Different Cohorts.

Model	Training Cohort		Validation Cohort		Immunotherapy Cohort		Multi‐omics Cohort	
	sensitivity	specificity	sensitivity	specificity	sensitivity	specificity	sensitivity	specificity
Fusion Clin‐SHR	87 (146/167)	87 (230/264)	77 (455/590)	83 (838/1005)	95 (52/55)	64 (21/33)	91 (84/92)	87 (63/73)
Fusion Clin‐TR	77 (129/167)	86 (228/264)	78 (461/590)	71 (713/1005)	91 (50/55)	55 (18/33)	85 (75/92)	67 (52/73)
Pre‐NAT SHR	71 (118/167)	87 (230/264)	75 (445/590)	65 (652/1005)	62 (34/55)	76 (25/33)	94 (82/92)	54 (41/73)
Pre‐NAT TR	70 (117/167)	78 (205/264)	69 (405/590)	62 (628/1005)	76 (42/55)	52 (17/33)	53 (50/92)	80 (56/73)
Mid‐NAT SHR	84 (141/167)	76 (200/264)	70 (414/590)	75 (753/1005)	87 (48/55)	58 (19/33)	88 (80/92)	64 (48/73)
Mid‐NAT TR	75 (126/167)	81 (215/264)	58 (345/590)	81 (811/1005)	82 (45/55)	67 (22/33)	80 (75/92)	67 (50/73)

Data are percentages, with proportions of patients (numerator/denominator) in parentheses; NAT = neoadjuvant treatment, SHR = spatial habitat radiomics, TR = traditional radiomics, AI = artificial intelligence.

### 2.4 Development of Multi‐Modality Models

Two multi‐modality models were developed:^[^ ^1^ ^]^ Clin‐SHR, integrating pre‐NAT and mid‐NAT SHR features with clinical parameters (cT stage, HER2 and ER status).^[^ ^2^ ^]^ Clin‐TR, combining pre‐NAT and mid‐NAT TR features with clinical parameters (cT stage, HER2, and ER status). In the training cohort, univariate and multivariate analysis identified high SHR scores on pre‐NAT and mid‐NAT MRI, cT1 stage, HER2‐positive and ER‐negative status as independent predictors of pCR (*p* <0.05, see Tables S2 and S3, Supporting Information). The model Clin‐SHR reached an AUC of 0.928 (95% CI 0.903 to 0.953) while the model Clin‐TR achieved an AUC of 0.879 (95% CI: 0.846, 0.912). The Clin‐SHR also exceeded Clin‐TR in sensitivity (87.43% vs 77.25%) and specificity (87.12% vs 86.36%) (Table 2). In the external validation cohort, Clin‐SHR also outperformed Clin‐TR, showing a higher AUC (0.863 versus 0.808), accuracy (81.07% vs 73.61%), and specificity (83.38% vs 70.95%). Clin‐SHR maintained this advantage in both the immunotherapy cohort and the multi‐omics cohort, particularly in specificity (63.64% vs 54.55% and 86.30% vs 68.49%, respectively). Decision curve analysis demonstrated that Clin‐SHR offered a greater net benefit than other models when the threshold probability ranged from 30% to 82%. To visually observe changes in intra‐tumor heterogeneity during NAT, we displayed a 3D view of spatial habitat from two representative patients (Figure S5, Supporting Information).

**Figure 4:**
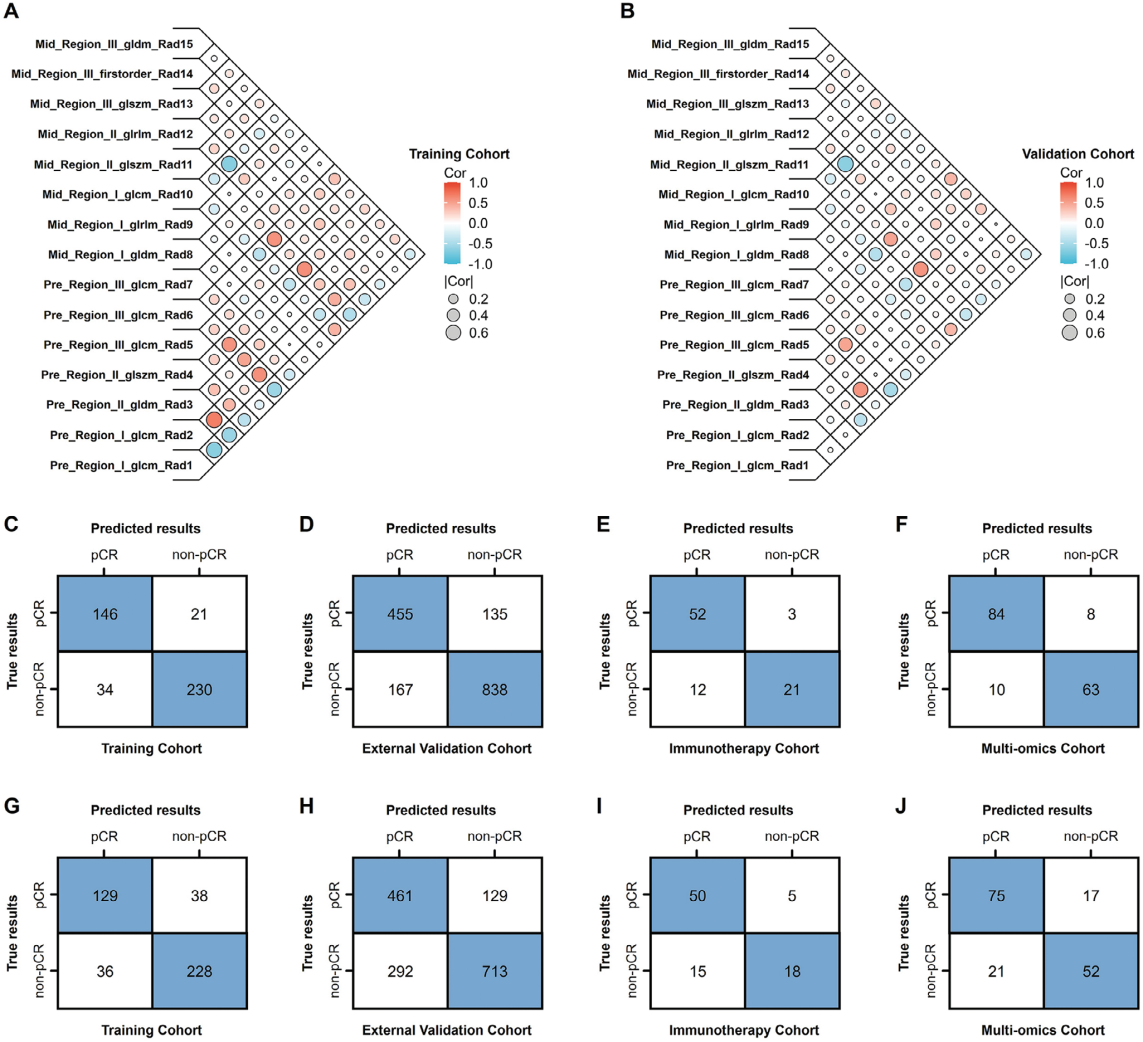
Spearman correlation coefficient matrices of selected imaging features and confusion matrices of predictive models. A,B) Spearman correlation coefficient matrices of selected radiomic features in the training and validation cohorts. C–F) Confusion matrices for the SHR model in the training, external validation, immunotherapy, and multi‐omics cohorts. G–J) Confusion matrices for the TR model in the training, external validation, immunotherapy, and multi‐omics cohorts. These results demonstrate the consistency of models' predictive performance and the radiomic features across different cohorts. pCR = pathological complete response.

The Spearman correlation coefficient matrices in **Figure** **4**A,B illustrate the relationship among imaging features in the training and validation cohorts, revealing consistent imaging patterns. The confusion matrices (Figure 4C–F) highlight the strong predictive power of Clin‐SHR across training, external validation, immunotherapy, and multi‐omics cohorts, underscoring its high accuracy in distinguishing pCR from non‐pCR. Clin‐TR (Figure 4G–J) also showed robust performance, confirming its reliability as a traditional radiomics model for predicting tumor response.

### 2.5 Clinical Subgroup Analysis to Validate Robustness of Multi‐Modality Models

Radiologists measured the maximum tumor diameter on MRI with high interobserver agreement (0.91 [95% CI: 0.84, 0.97] pre‐NAT; 0.88 [95% CI: 0.83, 0.93] mid‐NAT). Among 2279 patients, RECIST criteria indicated that 350 (15.36%) achieved a radiological complete response, while others had partial response, stable disease, or progression. For tumors that showed complete response on MRI, the Clin‐SHR model achieved a specificity of 88% and a positive predictive value of 93% for predicting pCR.

In validation cohorts, subgroup analysis showed consistent AUCs for the molecular subtypes (positive human epidermal growth factor receptor‐2 subtype [HER2+]: 0.84 [0.81, 0.87]; positive hormone receptor [HR] but negative HER2 subtype [HR+/HER2‐]: 0.86 [0.82, 0.90]; triple‐negative subtype [TN]: 0.86 [0.81, 0.91]) and clinical stages (stage II: 0.86 [0.83, 0.88]; stage III: 0.84 [0.81, 0.87]) (Table S4, Supporting Information). For the HER2+ subgroup, the Clin‐SHR reached an accuracy of 91.01% in the multi‐omics cohort, but it dropped to 78.84% in the validation cohort. For the HR+/HER2‐ subgroup, accuracy was consistent at 83.65%  in the validation cohort. The TN subgroup had lower overall accuracy (76.27% in the training versus 82.31% in the validation cohort) but showed higher sensitivity in the immunotherapy (94.55%) and multi‐omics (91.30%) cohorts. For clinical stages cTNM II and III, the Clin‐SHR demonstrated high accuracy in the training cohort (87.95% and 84.85%, respectively) and maintained strong performance in the multi‐omics cohort (87.80% and 92.86%, respectively), indicating robust performance across different clinical stages.

### 2.6 Radiomics Features Correlate with Tumoral Immune Microenvironment

**Figure 5:**
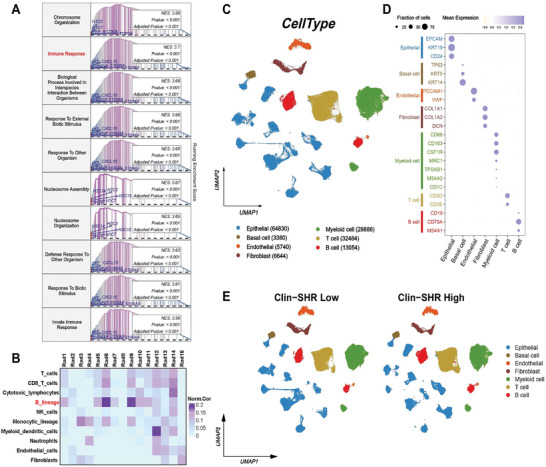
The associations between radiomics feature and immune landscape of low and high Clin‐SHR groups using transcriptomic and scRNA‐seq analysis. A) Gene set enrichment analysis highlights significant differences in biological processes, such as chromosome organization and immune response, between the two groups. B) A heatmap shows correlation between radiomics features and various immune cell populations. Each cell type is evaluated for its normalized correlation with specific radiomics features, indicating potential links between radiomics patterns and the tumor immune microenvironment. C) A UMAP visualization displays the distribution of different cell types, color‐coded for epithelial, basal, endothelial, fibroblast, myeloid, T cell, and B cell populations. D) The expression of key marker genes in each cell type, showing their average expression levels in the overall population. E) A UMAP visualization displays the distribution of different cell types, color‐coded for epithelial, basal, endothelial, fibroblast, myeloid, T cell, and B cell populations in low and high Clin‐SHR groups.

We analyzed bulk and single‐cell RNA‐seq data to investigate how radiomics features relate to the immune microenvironment and cell composition. We first measured Clin‐SHR levels in the RNA‐seq cohort, grouped patients into Clin‐SHR high or low, and conducted differential expression analysis to assess the potential biological basis of Clin‐SHR. Gene Set Enrichment Analysis (GSEA) showed that multiple immune response and chromosome organization processes were enriched in Clin‐SHR high patients (**Figure** **5**A). Notably, the immune response gene set had a normalized enrichment score (NES) of 3.7 (*p* <0.001), suggesting increased immune activity in Clin‐SHR high patients. We also observed enrichment in chromosome organization (NES = 3.88), nucleosome assembly (NES = 3.67), and nucleosome organization (NES = 3.65), indicating potential roles of chromatin remodeling and genome stability in this group. In addition, processes related to external biotic stimuli and defense responses (both NES = 3.68) were enriched, highlighting strong immune defense and interaction with external antigens in Clin‐SHR high patients. Next, we examined the correlation between imaging features and immune cell types (Figure 5B). A heatmap of 15 spatial habitat radiomics features showed that B cells were strongly correlated with most features, especially Rad5, Rad6, Rad9, Rad10, and Rad11 (correlation values: 0.15–0.3). T cells and cytotoxic lymphocytes had moderate correlations with Rad6, Rad10, and Rad14. These findings suggested that certain imaging patterns may indicate immune cell infiltration, which is important for monitoring tumor responses to NAT.

### 2.7 Single‐Cell Omics Analysis Uncovers Biological Basis of Multi‐Modality Model

We identified 46 cell clusters with moderate batch‐effect correction and preserved specificity of some cell subsets. Patients with different Clin‐SHR scores showed varying cell distributions (Figure S6, Supporting Information). Based on the expression of specific markers for the main cellular subpopulations in breast cancer samples (Figure 5D), we identified the aggregation of various cell populations (Figure 5C), including epithelial cells (EPCAM, KRT19, CD24), basal cells (TP63, KRT5, KRT14), endothelial cells (PECAM1, VWF), fibroblasts (COL1A1, COL1A2, and DCN), myeloid cells (CD68, CD163, and CSF1R), T cells (CD3D and CD3E) and B cells (CD19, CD79A, and MS4A1). Notably, B cells formed distinct clusters in the high Clin‐SHR group, suggesting higher B cell infiltration (Figure 5E). Independent UMAPs for each patient also showed varying B cell distributions (Figure S7, Supporting Information). Similar patterns emerged for T cells, indicating an overall increase in immune cell infiltration in patients with high Clin‐SHR scores.

**Figure 6:**
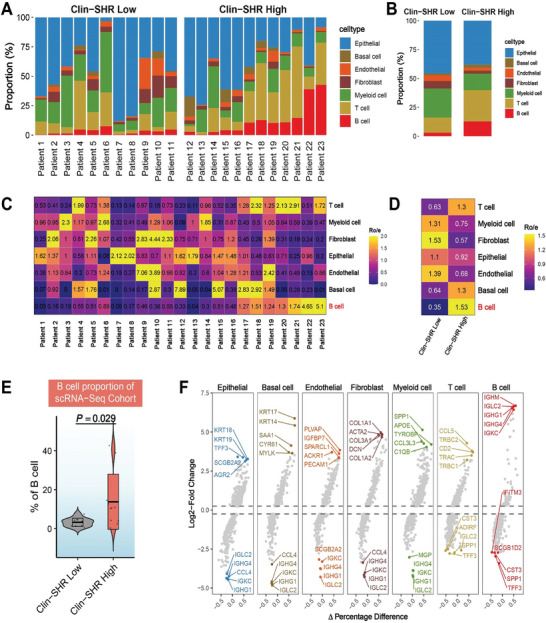
The difference of immune cell composition in high versus low Clin‐SHR scores using transcriptomic and scRNA‐seq analysis. A,B) The proportions of cell types between low and high Clin‐SHR patients. C,D) The ratio of observed over expected cell numbers (Ro/e) of different cell types between low and high Clin‐SHR patients. E) The proportion of B cell infiltration, with significant differences observed between low and high Clin‐SHR groups (P = 0.029). F) Differential gene expression profiles across cell types, with B cell‐specific genes prominently upregulated in high Clin‐SHR patients.

We compared cell‐type proportions in low and high Clin‐SHR scores subgroups and found that B cell fractions tended to rise as Clin‐SHR scores increased (**Figure** **6**A). In the high Clin‐SHR group, B cell proportions were higher than those in the low Clin‐SHR group (Figure 6B). To measure this more precisely, we used the ratio of observed to expected cell numbers (Ro/e), which showed that B cells gradually rose with increasing Clin‐SHR scores, reaching an average Ro/e of 1.53 in high Clin‐SHR tumors versus 0.46 in low Clin‐SHR tumors (Figure 6C,D). Violin plots confirmed higher B cell proportions in high Clin‐SHR patients (P = 0.029), and volcano plots revealed distinct gene expression in different cell subpopulations (Figure 6E,F). These results suggest that high Clin‐SHR tumors might have a more B cell‐driven immune microenvironment, which could affect tumor behavior and response to NAT.

**Figure 7:**
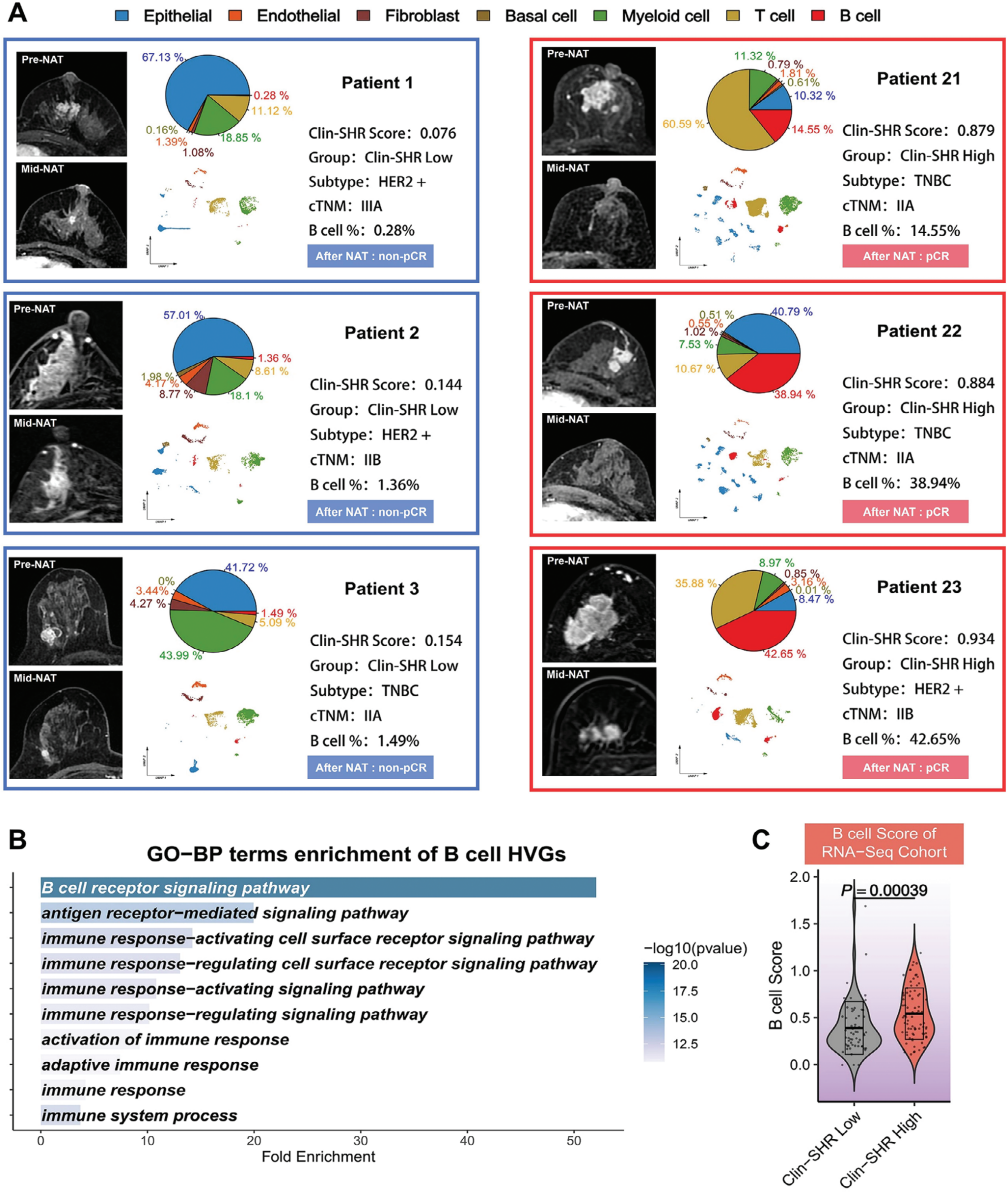
A comprehensive analysis of B cell infiltration in low and high Clin‐SHR breast cancer. A) Six representative patients’ pre‐NAT and mid‐NAT MRI images, alongside UMAP visualization depicting the cellular composition of their tumors. The pie charts detail the proportional representation of various cell types. B) The GO‐BP terms were enriched in highly variable genes (HVGs) of B cells, with the most significant pathways related to B cell receptor signaling and various immune response processes. C) The significant difference in B cell scores between low and high Clin‐SHR groups, with high Clin‐SHR patients showed higher B cell scores (p = 0.00039).

Next, we analyzed six representative patients (three pCR and three non‐pCR) for immune cell makeup and B cell enrichment, along with RNA‐seq data (*n* = 142). Among patients 1‐3 with low Clin‐SHR scores, B cell percentages were 0.28%, 1.36%, and 1.49%, respectively, whereas the patients 21‐23 with high Clin‐SHR scores showed significantly higher B cell levels (14.55%, 38.94%, and 42.65%, **Figure** **7**A). This pattern suggests a link between increased B cell infiltration and better treatment outcomes. Using highly variable genes (HVGs) derived from B cells, Gene Ontology enrichment revealed key pathways such as the B cell receptor signaling pathway and immune response‐activating signaling pathway, indicating the functional importance of B cells in immune activation and anti‐tumor responses (Figure 7B). We then used ssGSEA to generate a B cell score for each patient, which was significantly higher in the high Clin‐SHR group (P = 0.00039), suggesting a potential role for increased B cell infiltration in enhancing immune responses and treatment efficacy (Figure 7C).

## 3 Discussion

Radiomics models have the potential to improve breast cancer response stratification for patients receiving NAT. However, few studies have explored longitudinal MRI‐based spatial habitat radiomics for detecting changes in tumor heterogeneity and predicting outcomes. This study used a novel multi‐modality approach to more accurately predict pCR in breast cancer, showing the clinical value of combining pre‐NAT and mid‐NAT MRI scans. We found that cT1 stage, ER‐negative status, HER2‐positive status, and high SHR scores based on pre‐NAT and mid‐NAT MRI were independent predictors of pCR. In a large external validation cohort, the Clin‐SHR model outperformed single time‐point models and the Clin‐TR model (AUC: 0.85 vs 0.70–0.81; all p‐values < 0.05). Furthermore, Clin‐SHR model scores can reveal B cell infiltration and corresponding signaling pathway activation in a non‐invasive way.

Tumor heterogeneity, both between and within tumors, significantly affects treatment outcomes.^[^ ^28^ ^]^ However, the RECIST criteria focus only on the tumor's maximum diameter and cannot capture intra‐tumor heterogeneity. In our study, just 68% of patients who showed a radiological complete response at the midpoint of NAT achieved pCR. While advances in dynamic contrast‐enhanced MRI and hybrid imaging have improved prediction accuracy, they still rely on whole‐tumor analysis and may overlook tumor complexity.^[^ ^19^, ^29^, ^30^ ^]^ For example, a machine learning model that used pre‐treatment clinical and pathological features predicted pCR for breast cancer with an AUC of 0.81.^[^ ^31^ ^]^ Other studies using pre‐treatment imaging‐based radiomics reported AUCs from 0.80 to 0.85.^[^ ^32^, ^33^ ^]^ Similarly, convolutional neural networks trained on MRI data showed strong performance in predicting pCR of breast cancer, with AUCs above 0.79.^[^ ^34^, ^35^ ^]^ However, these methods often rely on baseline imaging or lack biological interpretation. In contrast, spatial habitat analysis focuses on features from distinct subregions within the tumor, rather than treating the tumor as a homogeneous entity.^[^ ^17^, ^18^ ^]^ This approach is based on the premise that intra‐tumor heterogeneity strongly influences treatment response and changes during NAT. Several studies have investigated the potential utility of spatial habitat analysis in predicting histological characteristics, treatment response, and survival outcomes in various cancers.^[^ ^36^, ^37^, ^38^, ^39^ ^]^ For breast cancer, spatial habitat analysis can capture the tumor's spatial heterogeneity.

When comparing our model to non‐traditional approaches, such as those based on radiomics or deep learning, the integration of multi‐modal data significantly enhances performance. Past work has shown that MRI‐based volume, histogram, and radiomics analyses can predict pCR in breast cancer, but with limited accuracy.^[^ ^19^, ^22^, ^23^, ^24^ ^]^ Another study has reported a radiomics model for predicting pCR using pre‐treatment images in breast cancer with AUCs of 0.76–0.82.^[^ ^29^ ^]^ Deep learning models, such as convolutional neural networks or transformer networks, have made progress in medical image analysis from tumor segmentation to treatment outcome prediction but often lack interpretability and struggle to integrate non‐imaging data.^[^ ^40^, ^41^, ^42^ ^]^ For instance, a study explored a solution for human‐AI collaboration for lymph node metastasis diagnosis based on medical images using a Transformer network.^[^ ^43^ ^]^ Our research highlights the clinical value of spatial habitat analysis, which extracts radiomics features from different tumor subregions instead of the entire tumor. A recent study showed that a habitat radiomics model predicted overall survival risk in esophageal squamous cell carcinoma, reaching a C‐index of 0.705.^[^ ^36^ ^]^ Another report found that space‐resolved radiomics and deep learning methods outperformed conventional radiomics for predicting breast cancer response to NAT.^[^ ^44^ ^]^ Consistent with those findings, we observed strong correlations between certain spatial habitat radiomics features and tumor outcomes. This suggests that when traditional radiomics fails to predict pCR, specific tumor subregions may provide critical information. This approach can guide personalized treatments by adjusting the NAT regimen and may help avoid overtreatment in patients with poor responses.

While spatial habitat analysis has introduced quantitative imaging biomarkers, studies often rely on single time‐point analyses, which fail to reflect dynamic changes during NAT,^[^ ^18^ ^]^ the subregional changes within tumors on longitudinal MRI remain unclear. Recent studies have used deep learning models to predict tumor response based on pathology images, achieving better performance than traditional clinical models.^[^ ^45^, ^46^ ^]^ A previous study also showed that longitudinal radiomics combined with deep learning methods have demonstrated great performance with AUCs of 0.837‐0.929 for predicting pCR.^[^ ^13^ ^]^ However, most imaging models focus on the whole tumor at a single time point, missing spatial and temporal variations. In contrast, we incorporated SHR features extracted from multiple time‐point MRIs and monitored their early changes during NAT. We also found that grade III tumors had higher pCR rates than lower‐grade tumors, likely due to faster cell growth, making them more sensitive to cytotoxic treatments. By combining imaging and clinical data, the Clin‐SHR model monitors how tumors evolve during NAT and helps decide whether the therapy is working. Spatial habitat analysis can detect changes in tumor intensity or texture before they become visible, allowing for earlier clinical adjustment. Meanwhile, changes in the radiomics feature map may show different risk groups in breast cancer, guiding closer follow‐up for patients with worse predictive results.

Furthermore, the inclusion of transcriptomics and scRNA‐seq data provides a molecular basis for the imaging model. While recent studies have used 50‐gene predictors or proteogenomic models to predict breast cancer treatment outcomes, they haven't included clinical imaging data.^[^ ^5^, ^47^ ^]^ In our study, the GSEA and MCPcounter results of the RNA‐seq cohort revealed that high Clin‐SHR scores were associated with increased activity in immune‐related pathways and immune cell index, particularly immune response activation and B cell infiltration. Spatial habitat analysis divides the tumor into different regions, helping us identify imaging features related to the tumor microenvironment. These imaging features may reflect immune cell infiltration, which involves changes in blood flow and the surrounding tissue.^[^ ^47^, ^48^ ^]^ Our scRNA‐seq analysis revealed that tumors with high Clin‐SHR scores have more B cell infiltration and higher expression of B cell‐related genes like IGHM, IGHG1, and IGHG2. This suggests that B cells play a key role in tumor response to NAT. This finding is consistent with previous studies showing that B cells support anti‐tumor immunity by producing antibodies and presenting antigens, enhancing the effectiveness of chemotherapy.^[^ ^49^, ^50^ ^]^ Moreover, higher Clin‐SHR scores may represent regions with active vasculature that recruit immune cells, including B cells, to activate an anti‐tumor response. The intensity, texture, and wavelet radiomics features capture differences in blood vessel structures, which are related to immune cell activity. B cell infiltration induces changes in the tumor microenvironment, such as increased extracellular matrix turnover and vascular remodeling, which can be detected by the intensity and texture features of SHR. These results suggest that targeting B cells can increase immune activity and improve NAT effectiveness, especially in HER2+ and TNBC breast cancer.^[^ ^51^, ^52^ ^]^ Further enrichment analysis of these modules indicated the biological relevance of these imaging features in capturing tumor immune heterogeneity.^[^ ^53^, ^54^, ^55^, ^56^ ^]^ These findings provide a clearer biological basis for our model and identify potential therapeutic targets for breast cancer.

The spatial habitat analysis utilized a predetermined algorithm, which has some limitations. Firstly, different segmentation methods may lead to different radiomics features, potentially affecting model performance. Secondly, incorporating more MRI sequences might help build a more comprehensive model. Lastly, while the model demonstrated favorable predictive ability, it requires further validation in prospective trials. In the future, integrating multi‐omics data such as proteomics and metabolomics can improve the model performance. For feature engineering, advanced techniques like deep learning‐based feature extraction and autoencoders can uncover relationships in multi‐omics data. In addition, the integration of patient‐specific clinical data such as comorbidities, treatment history, and genetic susceptibility can support more personalized assessments. Finally, to enhance the interpretability of the model, techniques such as SHAP and LIME can be used, enabling clinicians to understand the driving predictors behind the model.

In conclusion, our study develops a novel longitudinal MRI‐driven multi‐modality model using spatial habitat radiomics to predict pCR in breast cancer. The integration of multi‐time‐point MRI with spatial habitat radiomics represents a novel approach that enhances the predictive performance compared to traditional methods. Transcriptomic and single‐cell sequencing analysis revealed that the model can reflect the immune microenvironment, particularly B cell infiltration. Our findings offer a noninvasive tool for personalized treatment, addressing the critical need for early identification of pCR patients. However, the model requires further validation in prospective clinical trials to confirm its clinical value across different populations.

## 4 Experimental Section

### Study Design and Participants

From July 2015 to April 2023, 2256 breast cancer patients who underwent NAT and surgery (Supporting Information) were enrolled. Inclusion criteria were as follows: a) unilateral invasive breast cancer; b) undergoing pre‐NAT and mid‐NAT MRI scans; c) pathological assessment after NAT; and d) complete baseline data (Supporting Information). Exclusion criteria included the following: a) lack of MRI sequence or poor image quality; b) previous antineoplastic therapy; c) non‐standard NAT regimen; d) surgery at another center; and e) distant metastasis. This study followed the TRIPOD guidelines.^[^ ^57^ ^]^

### Human Specimens Acquisition

Fresh tumor samples were obtained from patients using ultrasound‐guided fine‐needle aspiration before treatment. RNA‐seq analysis was performed on samples from 142 patients (81 pCR and 61 non‐pCR), while scRNA‐seq analysis was conducted on samples from 23 patients (10 pCR and 13 non‐pCR). The study was approved by the hospital's Research Ethics Committee (Approval No. KY2023‐211‐01), and all participants provided written informed consent. Details on sample preparation and sequencing methods are available in the Supporting Information.

### MRI Protocol and Preprocessing

MRI images were acquired using 1.5‐T and 3.0‐T scanners, as detailed in Table S5 (Supporting Information). Dynamic contrast‐enhanced MRI sequences were collected for all patients, and image resolution was enhanced using a generative adversarial network. Preprocessing steps included N4 bias field correction to address field strength variations, B‐spline interpolation to standardize the spatial resolution to 1 mm^3^ isotropic voxel size, and histogram standardization to normalize intensity values for consistent comparisons. Further details are provided in Supporting Information.

### Pathological Assessment

Pathological evaluation of surgical specimens was conducted by specialized breast pathologists. Immunohistochemistry was performed on pre‐NAT biopsy samples to assess molecular markers, including estrogen receptor (ER), progesterone receptor (PR), human epidermal growth factor receptor‐2 (HER2), and the Ki‐67 index. For HER2 scores of 2+, fluorescence in situ hybridization was used to confirm gene amplification. After NAT, pCR was defined as the absence of invasive cancer in the breast and axillary lymph nodes, including ductal carcinoma in situ (ypT0/isN0). Patients were categorized into three subgroups based on HER2 and hormone receptor status:^[^ ^1^ ^]^ HER2+ subtype,^[^ ^2^ ^]^ HR+/HER2‐ subtype, and^[^ ^3^ ^]^ TN subtype.

### Identification of Tumor Subregions

Spatial habitat radiomics used imaging‐derived features to segment tumors into distinct subregions, or “habitats,” based on intra‐tumoral variations in perfusion, cellular density, and metabolic activity. These subregions were identified using K‐means clustering on dynamic contrast‐enhanced MRI images, reflecting varying biological processes, such as hypoxia, necrosis, or increasing cellular proliferation. Then habitat radiomics features are extracted from each subregion within the tumor. A two‐step pipeline was developed to identify tumor subregions. Three radiologists, unaware of any pathological findings, manually delineated the entire tumor using 3D Slicer software, covering both enhanced and necrotic areas (Figure S8, Supporting Information). In the training cohort, the primary tumor was segmented on pre‐NAT and mid‐NAT MRIs into super‐pixels. K‐means clustering was applied to the MRIs to delineate distinct tumor habitats, representing subregions with different perfusion characteristics. The optimal number of clusters was determined using the Calinski‐Harabasz score and validated through silhouette analysis.^[^ ^58^ ^]^ The resulting clusters were spatially mapped back onto the MRIs, creating a habitat map for each tumor (Supporting Information).

### Feature Extraction and Selection

The Image Biomarker Standardization Initiative guidelines were adhered to and extracted radiomics features from the whole tumor (traditional radiomics) and each tumor subregion (spatial habitat radiomics), including shape, intensity, texture, and wavelet features^[^ ^59^ ^]^ (Supporting Information). A consistent number of tumor subregions across pre‐NAT and mid‐NAT MRI scans were ensured. Only features with ICC greater than 0.8 for both intra‐ and inter‐reader reliability were retained, indicating high reproducibility.^[^ ^60^ ^]^

For model construction, a multistep feature selection process was used. First, features with low variance or high correlation (Spearman's correlation coefficient > 0.9) were excluded. Next, a univariate analysis using the Mann‐Whitney U test identified features significantly associated with pCR (*p* <0.05). To minimize overfitting, LASSO regression was applied. This process resulted in four independent feature sets: pre‐NAT TR, pre‐NAT SHR, mid‐NAT TR, and mid‐NAT SHR (Table S6, Supporting Information).

### Model Construction

In the training cohort, XGBoost models were developed using SHR or TR features to predict pCR. Model performance was assessed with tenfold cross‐validation, using the AUC as the primary evaluation metric (Supporting Information). The Clin‐SHR and Clin‐TR models were constructed by combining XGBoost models that integrated total SHR or TR features with significant clinical factors. Subgroup analysis was conducted to further validate the best‐performing model on different molecular subtypes and clinical TNM stages. All models were trained on a single NVIDIA RTX A6000 48GB GPU.

### Transcriptomic Analysis

Transcriptome data from RNA sequencing were processed to explore gene expression patterns in breast cancer. Gene expression was quantified using featureCounts and normalized by Transcripts Per Million (TPM) to adjust for library size differences. Differentially expressed genes (DEGs) between the high and low Clin‐SHR groups were identified using limma, with a p‐value threshold of 0.05. Gene set enrichment analysis (GSEA) was conducted on all eligible genes with clusterProfiler and org.Hs.eg.db, and results were visualized using GseaVis. Immune cell infiltration indices for all RNA‐seq samples were assessed with MCPcounter, and Spearman correlation analysis was performed between these indices and 15 radiomics features selected from a previous LASSO screen. Correlation coefficients were log‐normalized, and the results were visualized using pheatmap. Pathway enrichment analysis was performed with ClusterProfiler using Gene Ontology Biological Process (GO‐BP) terms, and pathways with significant enrichment were identified using an adjusted p‐value threshold of 0.05. Single‐sample GSEA (ssGSEA) was applied using GSVA with a B cell‐specific gene set to calculate the B cell score and evaluate B cell infiltration in the RNA‐seq cohort.

### Single‐Cell RNA Sequencing Analysis

Single‐cell RNA sequencing (scRNA‐seq) data were analyzed to explore immune cell populations in the tumor microenvironment. Sequencing reads were processed using the Cell Ranger pipeline (v3.1) and aligned to the GRCh38 human genome. Cells were then filtered based on unique molecular identifier (UMI) counts, removing low‐quality cells and doublets. Data normalization and dimensionality reduction were carried out with Seurat (v4.0). Cell types were identified through clustering with Uniform Manifold Approximation and Projection (UMAP) and annotated using canonical markers for epithelial cells, B cells, T cells, and other immune populations. Differential gene expression between the high and low Clin‐SHR groups was assessed with Seurat's FindMarkers function. Immune cell proportions were calculated and compared across patients, with the immune cell composition visualized using UMAP projections and violin plots.

### Statistical Analysis

Statistical analyses were performed using R software (version 4.0.3). Continuous data were presented as medians with interquartile ranges (IQRs), compared using Student's *t*‐test or Mann‐Whitney U test. Categorical data were represented as counts and percentages, compared using Pearson's χ^2^ test or Fisher's exact test. Model performance was assessed using the receiver operating characteristic (ROC) curve, the area under the ROC curve, and other diagnostic metrics. A decision curve analysis (DCA) was performed to evaluate the clinical usefulness of the imaging models in predicting pCR. All analyses were conducted using two‐tailed tests, with a p‐value below 0.05 deemed to indicate statistical significance.

### Ethics Approval Statement

The multicenter research adhered to the Declaration of Helsinki and received approval from the ethics committees of all involved institutions.

### Patient Consent Statement

All participants provided written consent after being informed.

## Conflict of Interest

The authors declare no conflict of interest.

## Author Contributions

D.G.G. and K.W. designed and concepted the study. W.L., T.Z., L.F.H., C.C.G., S.Q.W., Y.Z., and H.Q. collected the clinical and imaging data. Q.W., C.C.G., and X.Y.S. delineated the tumor regions. Y.H.H., Z.Y.S., and Y.L. analyzed the data. K.W., W.C.G., Z.Y.W., and Y.L. verified the data. Y.H.H., Z.Y.S., and W.L. wrote and revised the manuscript draft. All authors participated in the revision of the manuscript. All authors had access to the data and agreed to submit the manuscript for publication. The corresponding author K.W. affirmed that all listed authors meet authorship criteria.

## Supporting information



Supporting Information

## Data Availability

The data that support the findings of this study are available from the corresponding author upon reasonable request.
