# Supplementary material for: Longitudinal MRI‐Driven Multi‐Modality Approach for Predicting Pathological Complete Response and B Cell Infiltration in Breast Cancer
Source: Adv Sci (Weinh). 2025 Feb 7;12(12):2413702. doi: 10.1002/advs.202413702 (PMC11948082; doi:10.1002/advs.202413702)
Supplement: Supplementary file 1 — Supporting Information [file ADVS-12-2413702-s001.docx]

**Supporting Information**

**List of** **Supporting Information**

**Supporting Method 1.** Neoadjuvant Chemotherapy Regimen

**Supporting Method 2.** Baseline Data

**Supporting Method 3.** T1+DCE Scanning

**Supporting Method 4.** Generative Adversarial Networks and Imaging Preprocessing

**Supporting Method 5.** ROI Segmentation of Tumor

**Supporting Method 6.** Tumor Region Clustering to Subregions

**Supporting Method 7.** Feature Extraction

**Supporting Method 8.** Feature Selection

**Supporting Method 9.** Support Vector Machine and XGBoost

**Supporting Method 10.** RNA-Seq and Single-Cell RNA-Seq

**Supporting Method 11.** B Cell Gene Signature for ssGSEA Analysis

**Table S1.** Comparison of the AUCs among Different Models in the Training and Validation Cohort

**Table S2.** Stepwise Multivariable Logistic Regression Analysis of SHR Scores and Clinical Factors Associated with pCR

Table S3. Stepwise Multivariable Logistic Regression Analysis of TR Scores and Clinical Factors Associated with pCR

Table S4. The Subgroup Performances of Fusion Clinical-SHR Model for Predicting pCR in Training Cohort and Validation Cohort

Table S5. MR scanning parameters across different institutions

**Table S6.** Selected Subregional Habitat Radiomics Features of RNA-Seq Cohort

**Fig S1.** Plot of error versus lambda of pre-NAT model and mid-NAT model; the x-axis represents the logarithmic scale of the regularization parameter lambda, while the y-axis represents the mean square error of the model. As λ increases (from left to right), the mean square error of the model changes. The λ value corresponding to the minimum mean square error is the optimal number of features for LASSO feature selection. (A) showed the LASSO feature selection for pre-NAT SHR model, (B) showed the LASSO feature selection for pre-NAT TR model, (C) showed the LASSO feature selection for mid-NAT SHR model, and (D) showed the LASSO feature selection for mid-NAT TR model.

**Fig S2.** Feature coefficient map; At the optimal λ value, each non-zero feature has a corresponding coefficient, and the coefficient values corresponding to the habitat radiomics features. (A) showed the LASSO feature coefficient for pre-NAT SHR model, (B) showed the LASSO feature coefficient for pre-NAT TR model, (C) showed the LASSO feature coefficient for mid-NAT TR model, and (D) showed the LASSO feature coefficient for mid-NAT SHR model.

**Fig S3.** The spearman correlation coefficient among habitat radiomics features from (A) pre-NAT and (B) mid-NAT MRI image for predicting pathological complete response.

**Fig S4.** The spearman correlation coefficient among radiomics features and clinical characteristics from (A) pre-NAT and (B) mid-NAT MRI images for predicting pathological complete response.

**Fig S**5. Calibration curve analysis of clinical-SHR model (A and B) and clinical-TR model (C and D) in the training and external validation cohort.

**Fig S6.** The Uniform Manifold Approximation and Projection (UMAP) visualization of single-cell RNA samples in different groups and individual patients. Panel group shows a UMAP projection of cells from Clin-SHR high and low groups. Panel Sample displays the distribution of individual patients' cells. Panel Cluster illustrates the clustering of cells into 46 distinct groups, emphasizing the diversity of the tumor immune microenvironment.

**Fig S7.** The UMAP visualization of cell composition across individual patients. This figure presents UMAP plots of immune cell populations for 23 individual patients (HER2+: 9 patients; and TN: 14 patients), and each plot shows the distribution of various immune cell types.

**Fig S8.** the segmentation of tumor region at baseline DCE MRI image, and we delineated the entire tumor region for further analysis. During ROI segmentation, the necrosis, air and calcification area of the breast cancer were including as well. Figure (A) and (B) was from a patient (66 years old; HER2+ breast cancer; cT2 stage) who achieved pathological complete response after neoadjuvant chemotherapy, while Figure (C) and (D) was from a patient (48 years old; TN breast cancer; cT3 stage) who did not achieve pathological complete response after neoadjuvant chemotherapy.

**Supporting Method 1: Neoadjuvant Chemotherapy Regimen**

Due to the wide acceptance and standardization of NAT, relatively consistent regimens, and consistent inclusion criteria of breast cancer patient, we determined the patient recruitment period as July 2015 to October 2023. In our study, we strictly required all the retrospective multicenter data to adhere to the NAT guidelines. For the TNBC (n=420), 264 patients received AC followed by docetaxel (AC-T) regimen, 116 patients received platinum-contained regimen (TCb); For HR+/HER2- breast cancer (n=737), all the patients received AC-T regimen without neoadjuvant endocrine therapy; For HER2+ breast cancer (n=1034), 301 patients received AC-T combining with Trastuzumab with/without Pertuzumab regimen, and 733 patients received TCb combining with Trastuzumab with/without Pertuzumab regimen. Furthermore, 88 patients form the immunotherapy cohort received EC-TCb combined with Pembrolizumab regimen.

**Supporting Method 2: Baseline Data**

Clinical information, including age and menstrual status, was collected. Additionally, data regarding the preclinical stage (cT and cN stage), surgery dates and procedures, and pathologic response to neoadjuvant chemotherapy (NAT) in both the breast and axilla were gathered from institutional archives. The histopathologic details encompassed histologic type, tumor grade, Ki67 proliferation index, hormone receptor (HR) status, and human epidermal growth factor receptor 2 (HER2) status from biopsies conducted before neoadjuvant chemotherapy (NAT). HR status was determined based on estrogen receptor (ER) and progesterone receptor (PR) status via immunohistochemistry. HR- was defined as ER- and PR-, while HR+ was defined as ER+ and/or PR+. HER2+ status was established if there was 3+ positivity in immunohistochemistry or HER2 gene amplification in fluorescence in situ hybridization examination. The Ki67 index was categorized as low (<20%) or high (≥20%) expression using a cutoff value of 20%.

**Supporting Method 3:** **T1+DCE Scanning**

We required all patients to undergo MRI scan within two weeks before NAT, and after the completion of either 3 (TCb) or 4 (AC-T) cycle of NAT. We strictly set and recorded the scanning parameters in each center, and ensured that each patient's pre-NAT and mid-NAT MRIs are acquired under the same scanning parameters. The patients which we retrospectively recruited all underwent MRI scan using a 3.0-T or 1.5-T scanner with a dedicated breast coil. An initial T1WI pre-contrast scan was collected before T1+C images scanning, and then T1+C images were acquired as six post contrast scans at intervals of 60 seconds following the intravenous injection of gadolinium contrast agent. A gadolinium-based agent (Magnevist; Bayer Healthcare, Berlin, Germany) was injected using an MR imaging compatible power injector at a rate of 2 ml/s and at a dose of 0.2 ml/kg of body weight, followed by a 20-ml saline flush with high-pressure injector. The second phase of T1+C (around 120s after the intravenous injection of gadolinium contrast agent) was chose for further analysis in this study. The acquisition parameters of T1+C are shown in Table E1.

**Supporting Method 4: Generative Adversarial Networks and Imaging Preprocessing**

We used a 3D super-resolution reconstruction technique for medical images that utilizes a generative adversarial network (GAN) as its basic architecture. Super-resolution reconstruction is a technique that aims to improve the spatial resolution of an image beyond the physical limitations of the imaging system.

GANs are a type of deep learning model that consists of two networks: a generator network and a discriminator network. The generator network generates high-resolution images from low-resolution images, while the discriminator network distinguishes between real and generated images. The two networks are trained in an adversarial manner, where the generator network tries to generate images that can fool the discriminator network, and the discriminator network tries to distinguish between real and generated images. This adversarial training process helps the generator network to learn the mapping between low-resolution and high-resolution images. The dataset used to train the 3D super-resolution reconstruction technique consists of kinds of medical images from TCIA (https://www.cancerimagingarchive.net/). The images are preprocessed to remove noise and artifacts and to normalize the intensity values. The images are then divided into low-resolution and high-resolution pairs, where the low-resolution images are obtained by downsampling the high-resolution images. The pairs are used to train the GAN model.

The loss function used in the GAN model consists of three components: gradient loss, L1 loss, and perceptual loss. The gradient loss encourages the generated images to have similar gradient values as the high-resolution images. The L1 loss measures the pixel-wise difference between the generated and high-resolution images. The perceptual loss measures the difference between the feature representations of the generated and high-resolution images obtained from a pre-trained deep learning model. The combination of these loss functions helps to ensure that the generated images are visually similar with the high-resolution images.

The 3D super-resolution reconstruction technique has shown promising results in improving the spatial resolution of medical images. For example, it can increase the spatial resolution by 4 times while maintaining the original image size. This means that a pixel volume of 1x1x4mm can be transformed into 1x1x1mm. The technique has been evaluated on various medical imaging modalities such as CT, MRI, and ultrasound, and has shown significant improvement in image quality and spatial resolution. The technique has also been compared with other state-of-the-art super-resolution reconstruction techniques and has shown superior performance. We wrote a medical image processing program in python, which can be used for various super-resolution reconstruction tasks of medical images (https://gitee.com/wangqingbaidu/OnekeyCompo/tree/master).

For each MRI sequence, N4ITK MRI bias correction and rotation to volume plane were done. We perform N4ITK MRI bias correction with N4 algorithm in 3D Slicer software (version 4.10.2, www.slicer.org). This algorithm is based on the ITK filters contributed in the following publication: Tustison N, Gee J "N4ITK: Nick's N3 ITK Implementation for MRI Bias Field Correction" (which is introduced in detail at http://hdl.handle.net/10380/3053), and the rotation to volume plane was performed using classical module in 3D Slicer software (version 4.10.2, www.slicer.org).

Since the intensity values of MR images distribute widely, we used z-score normalization to make the image intensities have the properties of a standard normal distribution with μ=1 and σ=0, where μ is the mean value of the intensities, and σ is the standard deviation. The normalized values (also called z scores) of the image intensities (x) were calculated as follows: z = (x-μ)/σ.

**Supporting Method 5: ROI Segmentation of Tumor**

The segmentation module in the 3D Slicer software (version 4.10.2, www.slicer.org) did the preliminary semi-automatic segmentation according to intensity threshold segmentation. The module is meant to create easy and effecient segmentations on high slice resolution medical images. It can calculate subtraction maps, register images, normalize images, create 3D volumetric ROIs using Delaunay Triangulation, and finally threshold intensities within an ROI. Then the manual corrections such as relabeling and holes filling were done by two professional radiologists with more than ten years of experience in consensus.

**Supporting Method 6: Tumor Region Clustering to Subregions**

To extract intra-tumor subregions within tumor objectively and avoid human bias, we applied a two-step pipeline. Firstly, three-dimensional tumor regions were delineated on original MRIs. In this study, a manual delineation approach was used via 3D slicer software (an opensource DICOM viewer, version 4.10.2). Secondly, considering efficiency, performance, and generalization, we used K-means clustering method segment super-voxels within intratumor. K-means is a popular clustering algorithm that is widely used in image processing and machine learning. In the context of MRI, K-means can be applied to segment different tissues or structures by grouping pixels into distinct clusters based on their intensity values. For each pixel in the image, The K-means method calculates its distance to all cluster centers (often using Euclidean distance) and assign it to the cluster corresponding to the nearest center. Recalculating the center of each cluster as the mean of all pixels currently assigned to that cluster until convergence, which occurs when the cluster assignments no longer change significantly or after a predefined number of iterations. In this study, we expected that tumors can be segmented into small regions initially, then grouped together based on further process, hence each tumor was segmented into subregions with a predefined parameter of segment number (n = 10). However, conventional radiomics extraction may fail in subregions with limited number of voxels. To solve this issue, a minimal volume threshold [1,1,1] was set.

The Calinski-Harabasz index, also known as the Variance Ratio Criterion (VRC), is a metric used to evaluate the quality of a clustering. The index is designed to measure the separation between clusters as well as the cohesion within clusters, with higher values indicating better defined clusters. The Calinski-Harabasz index is calculated using the following formula:

$$CH(k)=\frac{(B(k)/(k-1))}{(W(k)/(n-k))}$$

(1) $CH(k)$ is the Calinski-Harabasz index for *k* clusters

(2) $B\left( k \right)$ is the between-group variance (i.e., the variance between the different clusters).

(3) $W(k)$ is the within-group variance (i.e., the variance within each cluster).

(4) $n$ is the number of data points.

In simpler terms, the index is a ratio of the variance between clusters to the variance within clusters, normalized by the number of clusters and the number of data points. A higher *CH* index indicates that the clusters are well-separated and compact, which is generally desirable. The Calinski-Harabasz index provides a quantitative measure, but qualitative assessment and domain expertise are crucial for making the final decision. While the Calinski-Harabasz index is a useful tool for evaluating clustering results, it has some limitations. It may not perform well for datasets with clusters of varying densities or non-convex shapes. Additionally, like other clustering evaluation metrics, it should be used in conjunction with domain knowledge and other evaluation methods to ensure robust results.

**Supporting Method 7: Feature Extraction**

Before features extraction, the voxel size of each sequence was resampled to 1×1×1 mm^3^ and the bin width of gray-level histogram was fixed as 25. After z-score normalization of image pixel intensities, a total of 1223 quantitative imaging features including 8 shape-based features, 17 first order statistical features, 75 textural features from original images and 1123 derived features (706 features of Gabor-bank wavelet filtered images and 417 features of Law’s filtered images), were extracted T1+C images using corresponding ROIs.

**Supporting Method 8: Feature Selection**

To decrease the chance of overfitting, feature dimension reduction including (1) reproducibility assessment of habitat radiomics features, (2) Mann-Whitney U test statistical assessment, (3) Spearman correlation analysis, and (4) least absolute shrinkage and selection operator (LASSO).

**(1) reproducibility assessment using Intraclass Correlation Coefficient (ICC)**

Radiomics is a field in medical imaging that involves extracting lots of quantitative features from medical images, such as MRI or CT scans, to aid in diagnosis, prognosis, or treatment planning. The reproducibility of radiomics features is crucial to ensure that the results are reliable and can be replicated across different settings, patients, and imaging devices. Reproducibility assessment in radiomics refers to the evaluation of the consistency and stability of extracted features from medical images. It aims to determine whether the same features can be reliably extracted under different conditions, such as:

(i) Inter-Observer Variability: Variation in feature values when different observers delineate the region of interest (ROI).

(ii) Intra-Observer Variability: Variation in feature values when the same observer delineates the ROI at different times.

The ICC is a measure that assesses the consistency or agreement of quantitative measurements made by different observers measuring the same quantity. Values range from 0 (no agreement) to 1 (perfect agreement), with values > 0.8 generally considered good agreement. We used inter-reader and intra-reader ICC to select the robust habitat radiomics features for further analysis. For inter-reader ICC assessment, we randomly selected 200 patients in the training cohort, and assigned one radiologist to delineate all the tumor regions. After one month, the same radiologist delineated all the tumor regions of those 200 patients again, and then habitat radiomics features were extracted and calculated the ICC value, and only the features with an ICC value > 0.8 were retained. For intra-reader ICC assessment, we randomly selected 200 patients in the training cohort, and assigned two radiologists to delineate all the tumor regions, and then habitat radiomics features were extracted and calculated the ICC score, and only the features with an ICC value > 0.8 were retained.

**(2) Mann-Whitney U test**

The Mann-Whitney U test (also known as the Wilcoxon rank-sum test) is a non-parametric statistical test used to determine whether there is a significant difference between the distributions of two independent groups. In radiomics, the Mann-Whitney U test can be used to compare the radiomic features extracted from different groups of medical images, such as images from healthy versus diseased patients, or images before and after treatment. Below is the Mann-Whitney U statistic test for each group is calculated using the formula:

$$U=n_{1}\cdot n_{2}+\frac{n_{1}\cdot(n_{1}+1)}{2}-R_{1}$$

(i) $n_{1}$ and $n_{2}$ are the sample sizes of the two groups.

(ii) $R_{1}$ is the sum of the ranks for group 1.

**(3) Spearman correlation analysis**

Spearman correlation analysis is a non-parametric statistical test used to measure the strength and direction of association between two ranked variables. In radiomics, which involves the extraction of quantitative features from medical images to characterize tumor phenotype, Spearman correlation can be used to assess relationships between different radiomic features or between radiomic features and clinical parameters. The formula for Spearman’s rank correlation coefficient is:

$$r_{s}=1-\frac{6\sum d_{i}^{2}}{n(n^{2}-1)}$$

(i) $d_{i}^{2}$ is the difference between the ranks of corresponding variables.

(ii) $n$ is the difference between the ranks of corresponding variables.

Compute $r_{s}$​ for each pair of radiomic features or between radiomic features and clinical parameters. The closer absolute value of $r_{s}$ is to 1, the stronger the association.

**(4) LASSO (least absolute shrinkage and selection operator)**

The Lasso is a linear model that estimates sparse coefficients. It is useful in some contexts due to its tendency to prefer solutions with fewer non-zero coefficients, effectively reducing the number of features upon which the given solution is dependent. For this reason, Lasso and its variants are fundamental to the field of compressed sensing. Under certain conditions, it can recover the exact set of non-zero coefficients. As the Lasso regression yields sparse models, it can thus be used to perform feature selection, as detailed in L1-based feature selection.

**Supporting Method 9: Support Vector Machine and XGBoost**

SVM mainly used to solve data classification problems in the field of pattern recognition, which belongs to a kind of supervised learning algorithm. It has strong nonlinear classification ability, showing many unique advantages in solving small sample, nonlinear and high-dimensional pattern recognition problems. “C” and “gamma” were used in the tuning step of model development. XGBoost is a powerful machine learning algorithm based on decision trees, designed for speed and performance. It is an implementation of the gradient boosting framework that builds models sequentially, with each new model correcting the errors of the previous ones. XGBoost optimizes both bias and variance, leading to highly accurate predictions.

**Supporting Method 10: RNA-Seq and Single-Cell RNA-Seq**

1. **RNA-Seq**

The RNA extraction, mRNA purification, cDNA library quantification, and sequencing were all carried out according to the manufacturer's instructions and using commercial kits and equipment from reputable suppliers. After sequencing, the quality of the generated cDNA libraries was assessed using the Qubit dsDNA HS Assay Kit and Qubit 3.0 fluorometer. The obtained 150 bp paired-end reads were then processed using various bioinformatics tools such as FastQC, hisat2 and featureCounts to obtain a read-count matrix.

1. **Single-Cell RNA-Seq**

Tumor samples were obtained using ultrasound-guided fine needle aspiration (FNA). During the puncture procedure, it was ensured that aseptic handling was used to minimize sample contamination. The collected tissue block was then immediately placed in saline buffer to minimize tissue hypoxia and degradation. The mobile sample was brought to ice and sent to the laboratory for processing as soon as possible. In a sterile environment, biopsy samples were transferred to sterile centrifuge tubes containing cold PBS, and the tissue blocks were cut into small pieces (1-2 mm) with a scalpel, added to a digestive enzyme solution containing 1 mg/mL of collagenase IV and 0.1 mg/mL of DNA enzyme, and digested for 1 hour at 37°C in a warm oven, with regular gentle stirring to facilitate tissue digestion. The digested solution was filtered using a 70 μm cell sieve to remove undigested tissue mass, and then the cell sediment was separated by centrifugation at 300-500 g for 5-10 min. The supernatant was removed and the cell precipitate was resuspended in PBS to ensure a homogeneous cell suspension. Cells were counted and activity assayed using a Trypan Blue cell counting plate to ensure that cell viability was above 80%. The cell concentration was adjusted to the recommended concentration (1000-5000 cells/μL). Subsequently, single-cell library construction was completed using the Matrix NEO® Automated Single-Cell Sequencing Library Construction System. The single-cell RNA matrix was generated by CeleScope on Lunix.

**Supporting Method 11: B Cell Gene Signature for ssGSEA Analysis**

We used single-sample Gene Set Enrichment Analysis to represent the B cell signature. The extracted B cell-associated genes are upregulated highly variable genes (HVGs) of B cell in scRNA-Seq data, include IGHM, IGLC2, IGHG1, IGHG4, IGKC, IGHG3, IGLC3, IGHA1, IGHGP, JCHAIN, IGHG2, IGLV1-47, MZB1, IGLV2-14, MS4A1, DERL3, CD79A, SSR4, HERPUD1, IGLV3-1, CD79B, FKBP11, ITM2C, CD37, SEC11C, EAF2, RALGPS2, PIM2, MEF2C, FCRL5, NR4A1, SLAMF7, KLF2, TNFRSF17, ST6GAL1, POU2AF1, SEL1L3, TENT5C, JSRP1, BASP1, HSH2D, P2RX1, CYBA, BIRC3, FCHSD2, BLNK, FKBP2, TAGAP, SPCS3, UBE2J1, SDF2L1, DNAJB9, CD38, PRDX4, SELENOK, SEL1L, SPAG4, SDC1, SYVN1, CYTIP, ISG20, TXNDC11, FBXW7, ELL2, IL16, CD27, PLPP5, ICAM3, CPEB4, ICAM2, TXNDC15. These genes are key markers for B cell activity and are involved in processes such as immunoglobulin production, B cell receptor signaling, and immune regulation, providing insights into the immune landscape in cancer. These genes are essential markers reflecting the level of B cell infiltration and activity in the tumor microenvironment, providing insights into the immune landscape and potential therapeutic responses in cancer patients.

Table S1**.** **Comparison of the AUCs among Different Models in the Training and Validation Cohort**

| Model | Training Cohort (n=431) | | Validation Cohorts (n=1848) | |
| --- | --- | --- | --- | --- |
|  | AUC (95% CI) | p-value | AUC (95% CI) | p-value |
| Fusion Clinical-SHR | 0.92 (0.90, 0.95) | reference | 0.85 (0.83, 0.87) | reference |
| Fusion Clinical-TR | 0.88 (0.85, 0.91) | **.045*** | 0.81 (0.79, 0.83) | **.004*** |
| Pre-NAT SHR | 0.83 (0.79, 0.87) | **<.001*** | 0.75 (0.73, 0.78) | **<.001*** |
| Mid-NAT SHR | 0.87 (0.84, 0.90) | **.004*** | 0.79 (0.76, 0.81) | **<.001*** |
| Pre-NAT TR | 0.82 (0.78, 0.85) | **<.001*** | 0.70 (0.68, 0.73) | **<.001*** |
| Mid-NAT TR | 0.83 (0.80, 0.87) | **<.001*** | 0.76 (0.73, 0.78) | **<.001*** |

Note. - the p-value represented the DeLong test on AUCs between models. AUC = the area under the curve; NAT = neoadjuvant chemotherapy; SHR = spatial habitat radiomics; TR = traditional radiomics; CI = confident interval.

***** Indicates statistical significance; *p* value < .05.

| Factor | Multivariate Analysis | | |
| --- | --- | --- | --- |
|  | β Coefficient | Odds Ratio (95% CI) | P value |
| Pre-NAT SHR score |  |  |  |
| Low | Reference |  |  |
| High | 2.61 | 13.63 (7.31, 25.40) | **<.001*** |
| Mid-NAT SHR score |  |  |  |
| Low | Reference |  |  |
| High | 2.84 | 17.10 (9.03, 32.39) | **<.001*** |
| Pre-treatment cT stage |  |  |  |
| cT1 | Reference |  |  |
| cT2 | -1.34 | 0.26 (0.08, 0.87) | **.029** |
| cT3 | -1.57 | 0.21 (0.05, 0.81) | **.024** |
| cT4 | -2.83 | 0.06 (0.01, 0.36) | **.002** |
| HER2 status |  |  |  |
| Negative | Reference |  |  |
| Positive | 1.70 | 5.47 (3.03, 9.87) | **<.001*** |
| ER status |  |  |  |
| Negative | Reference |  |  |
| Positive | -1.36 | 0.26 (0.10, 0.70) | **.04*** |

Table S2**.** **Stepwise Multivariable Logistic Regression Analysis of SHR Scores and Clinical Factors Associated with pCR**

Note. - *****Indicates statistical significance; *p* value < .05. ER = estrogen receptor; HER2 = human epidermal growth factor receptor 2; pCR = pathological complete response; CI = confident interval; TR = traditional radiomics.

***** Indicates statistical significance; *p* value < .05.

| Factor | Multivariate Analysis | | |
| --- | --- | --- | --- |
|  | β Coefficient | Odds Ratio (95% CI) | P value |
| Pre-NAT TR score |  |  |  |
| Low score | Reference |  |  |
| High score | 2.03 | 7.64 (4.31, 13.54) | **<.001*** |
| Mid-NAT TR score |  |  |  |
| Low score | Reference |  |  |
| High score | 2.72 | 15.25 (8.46, 27.47) | **<.001*** |
| Pre-treatment cT stage |  |  |  |
| cT1 | Reference |  |  |
| cT2 | -0.65 | 0.52 (0.18, 1.51) | .232 |
| cT3 | -0.78 | 0.46 (0.13, 1.59) | .218 |
| cT4 | -2.13 | 0.12 (0.02, 0.65) | **.014*** |
| HER2 status |  |  |  |
| Negative | Reference |  |  |
| Positive | 1.84 | 6.29 (3.57, 11.07) | **<.001*** |
| ER status |  |  |  |
| Negative | Reference |  |  |
| Positive | -1.42 | 0.24 (0.09, 0.63) | **.004*** |

Table S3**.** **Stepwise Multivariable Logistic Regression Analysis of TR Scores and Clinical Factors Associated with pCR**

Note. - *****Indicates statistical significance; *p* value < .05. ER = estrogen receptor; HER2 = human epidermal growth factor receptor 2; pCR = pathological complete response; CI = confident interval; TR = traditional radiomics.

***** Indicates statistical significance; *p* value < .05.

| Subgroup | Cohort | AUC (95% CI) | ACC (%) | SEN (%) | SPE (%) | PPV (%) | NPV (%) |
| --- | --- | --- | --- | --- | --- | --- | --- |
| HER2+ | TC | 0.96 (0.74, 0.98) | 87.89 | 84.97 | 94.29 | 97.01 | 74.16 |
|  | VC | 0.84 (0.81, 0.87) | 77.42 | 75.00 | 80.11 | 80.74 | 74.24 |
| HR+/HER2- | TC | 0.92 (0.87, 0.97) | 88.07 | 88.89 | 87.96 | 51.06 | 98.25 |
|  | VC | 0.86 (0.82, 0.90) | 83.27 | 74.63 | 84.53 | 41.32 | 95.80 |
| TN | TC | 0.83 (0.72, 0.94) | 80.28 | 78.57 | 82.76 | 86.84 | 72.73 |
|  | VC | 0.86 (0.81, 0.91) | 81.29 | 79.13 | 82.68 | 74.59 | 86.05 |
| TNM stage II | TC | 0.91 (0.88, 0.94) | 86.70 | 82.95 | 89.77 | 86.90 | 86.55 |
|  | VC | 0.86 (0.83, 0.88) | 80.43 | 77.51 | 82.57 | 76.47 | 83.40 |
| TNM stage III | TC | 0.95 (0.92, 0.99) | 87.60 | 89.13 | 86.67 | 80.39 | 92.86 |
|  | VC | 0.84 (0.81, 0.87) | 79.61 | 72.85 | 82.60 | 64.92 | 87.32 |
| rCR type | TC | 0.91 (0.83, 0.99) | 85.92 | 84.44 | 88.46 | 92.68 | 76.67 |
|  | VC | 0.87 (0.82, 0.92) | 79.92 | 76.88 | 86.84 | 93.01 | 62.26 |

Table S4. The Subgroup **Performances of Fusion Clinical-SHR Model for Predicting pCR in Training Cohort and Validation Cohort**

Note. - Unless otherwise specified, data are percentages; NAT = neoadjuvant chemotherapy; TN = triple negative; HER2 = human epidermal growth factor receptor 2; HR = hormone receptor; TC = training cohort; VC = validation cohort; AUC = the area under the curve; ACC = accuracy; SEN = sensitivity; SPE = specificity; PPV = positive predicted value; NPV = negative predicted value; SHR = spatial habitat radiomics; CI = confidence interval

Table S5. MR Scanning Parameters across Different Institutions

| Cohort | Scanner | Sequence | TR/TE (ms) | FOV (mm) | Matrix | Slice Thickness (mm) | Slice Gap (mm) | Flip Angle |
| --- | --- | --- | --- | --- | --- | --- | --- | --- |
| I | Philips 1.5T (Achieva) | T1+C | 5.4/2.4 | 300×320 | 300×320 | 1.0 | 0 | 15° |
| II | Siemens 1.5T (Avanto) | T1+C | 4.43/1.5 | 340×340 | 448×336 | 1.7 | 0.34 | 10° |
| III | GE 1.5T (Signa HDx) | T1+C | 6.5/2.1 | 380×342 | 256×256 | 3.0 | 0 | 10° |
| IV | Siemens 3.0T (Skyra) | T1+C | 5.65/2.46 | 360×360 | 384×384 | 2.5 | 0.5 | 15° |
| V | GE 1.5T (Optima) | T1+C | 5.2/2.15 | 340×340 | 256×244 | 1.5 | 0.7 | 12° |
| VI | Siemens 3.0T (Skyra) | T1+C | 3.6/1.25 | 380×380 | 320×320 | 2.0 | 0.5 | 10° |
| VII | Philips 3.0T (Ingenia) | T1+C | 4.8/2.1 | 280×340 | 280×339 | 1.0 | 0 | 12° |
| VIII | GE 3.0T (Signa HDx) | T1+C | 5.6/2.2 | 380×380 | 320×320 | 3.0 | 0 | 10° |
| IX | Philip 3.0T (Ingenia) | T1+C | 4.3/2.1 | 280×339 | 280×337 | 2.0 | 0.6 | 12° |
| X | GE 3.0T (Signa HDx) | T1+C | 5.0/1.9 | 240×240 | 288×192 | 3.8 | 0 | 10° |
| XI | Siemens 1.5T (Avanto) | T1+C | 5.2/2.4 | 300×300 | 256×256 | 1.7 | 0.5 | 10° |
| XII | Philips 1.5T (Achieva) | T1+C | 5.0/2.2 | 360×360 | 320×320 | 1.5 | 0.3 | 12° |

Note. - The institusions I was used as the training cohort, and institusions II-XII were used as the validation cohort.

Table S6. Selected Subregional Habitat Radiomics Features (RNA-Seq)

| Feature Number | Feature Detail |
| --- | --- |
| Rad1 | Pre_Region_III_wavelet-LHL_glcm_InverseVariance |
| Rad2 | Pre_Region_I_wavelet-LLH_glcm_ClusterShade |
| Rad3 | Pre_Region_II_wavelet-LHH_gldm_DependenceNonUniformityNormalized |
| Rad4 | Pre_Region_I_wavelet-HLL_glcm_InverseVariance |
| Rad5 | Pre_Region_III_log-sigma-3_glcm_ClusterShade |
| Rad6 | Pre_Region_III_wavelet-LHH_glcm_ClusterTendency |
| Rad7 | Pre_Region_II_wavelet-LHL_glszm_LargeAreaLowGrayLevelEmphasis |
| Rad8 | Mid_Region_III_wavelet-HLH_gldm_LargeDependenceLowGrayLevelEmphasis |
| Rad9 | Mid_Region_II_log-sigma-4_glszm_GrayLevelNonUniformityNormalized |
| Rad10 | Mid_Region_I_log-sigma-1_gldm_LargeDependenceHighGrayLevelEmphasis |
| Rad11 | Mid_Region_III_log-sigma-4_glszm_SmallAreaLowGrayLevelEmphasis |
| Rad12 | Mid_Region_I_wavelet-HHH_glrlm_ShortRunEmphasis |
| Rad13 | Mid_Region_II_log-sigma-3_glrlm_ShortRunLowGrayLevelEmphasis |
| Rad14 | Mid_Region_III_log-sigma-4_firstorder_10Percentile |
| Rad15 | Mid_Region_I_wavelet-HLH_glcm_InverseVariance |


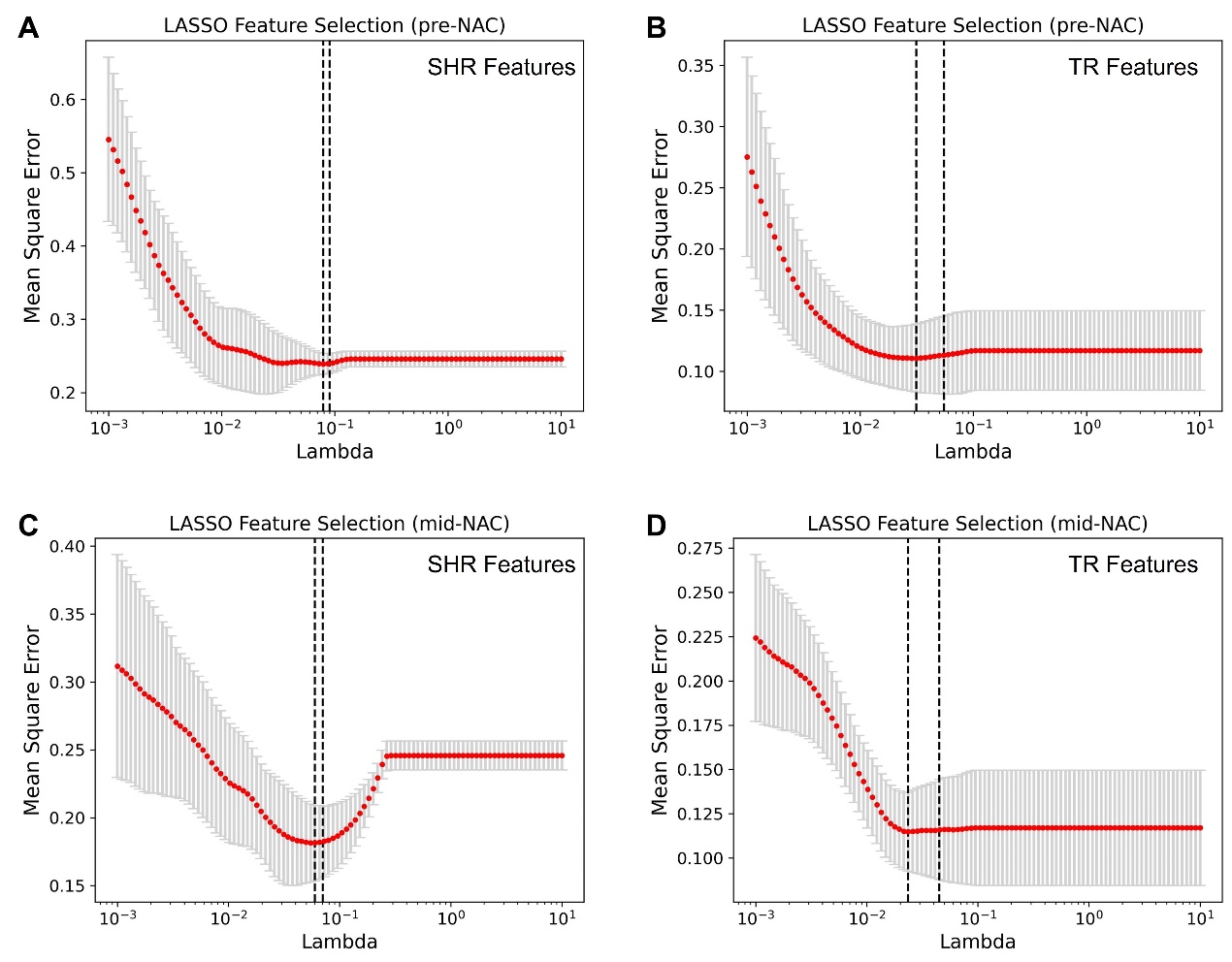


**Fig S1.** Plot of error versus lambda of pre-NAT model and mid-NAT model; the x-axis represents the logarithmic scale of the regularization parameter lambda, while the y-axis represents the mean square error of the model. As λ increases (from left to right), the mean square error of the model changes. The λ value corresponding to the minimum mean square error is the optimal number of features for LASSO feature selection. (A) showed the LASSO feature selection for pre-NAT SHR model, (B) showed the LASSO feature selection for pre-NAT TR model, (C) showed the LASSO feature selection for mid-NAT SHR model, and (D) showed the LASSO feature selection for mid-NAT TR model.


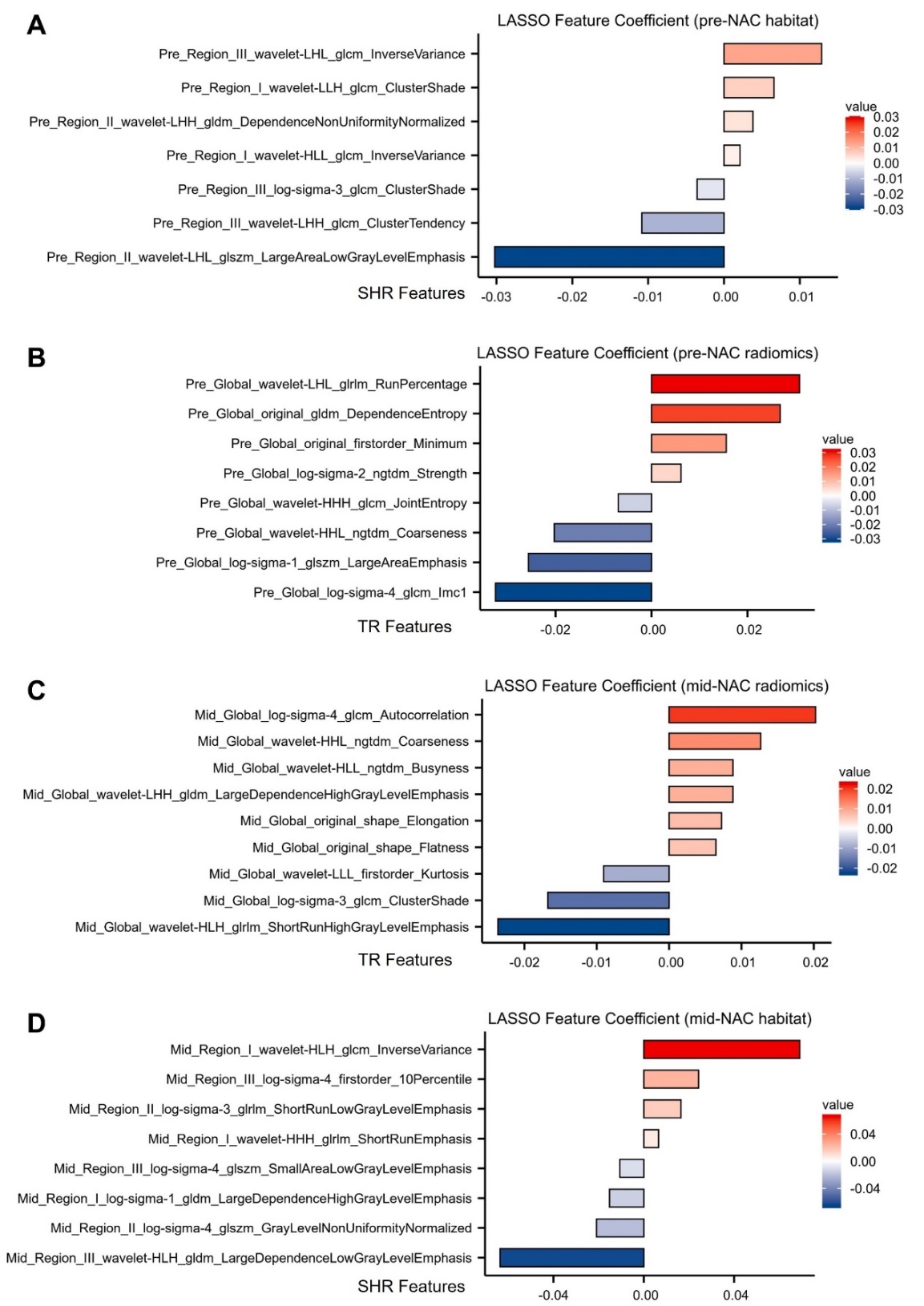


**Fig S2.** Feature coefficient map; At the optimal λ value, each non-zero feature has a corresponding coefficient, and the coefficient values corresponding to the habitat radiomics features. (A) showed the LASSO feature coefficient for pre-NAT SHR model, (B) showed the LASSO feature coefficient for pre-NAT TR model, (C) showed the LASSO feature coefficient for mid-NAT TR model, and (D) showed the LASSO feature coefficient for mid-NAT SHR model.


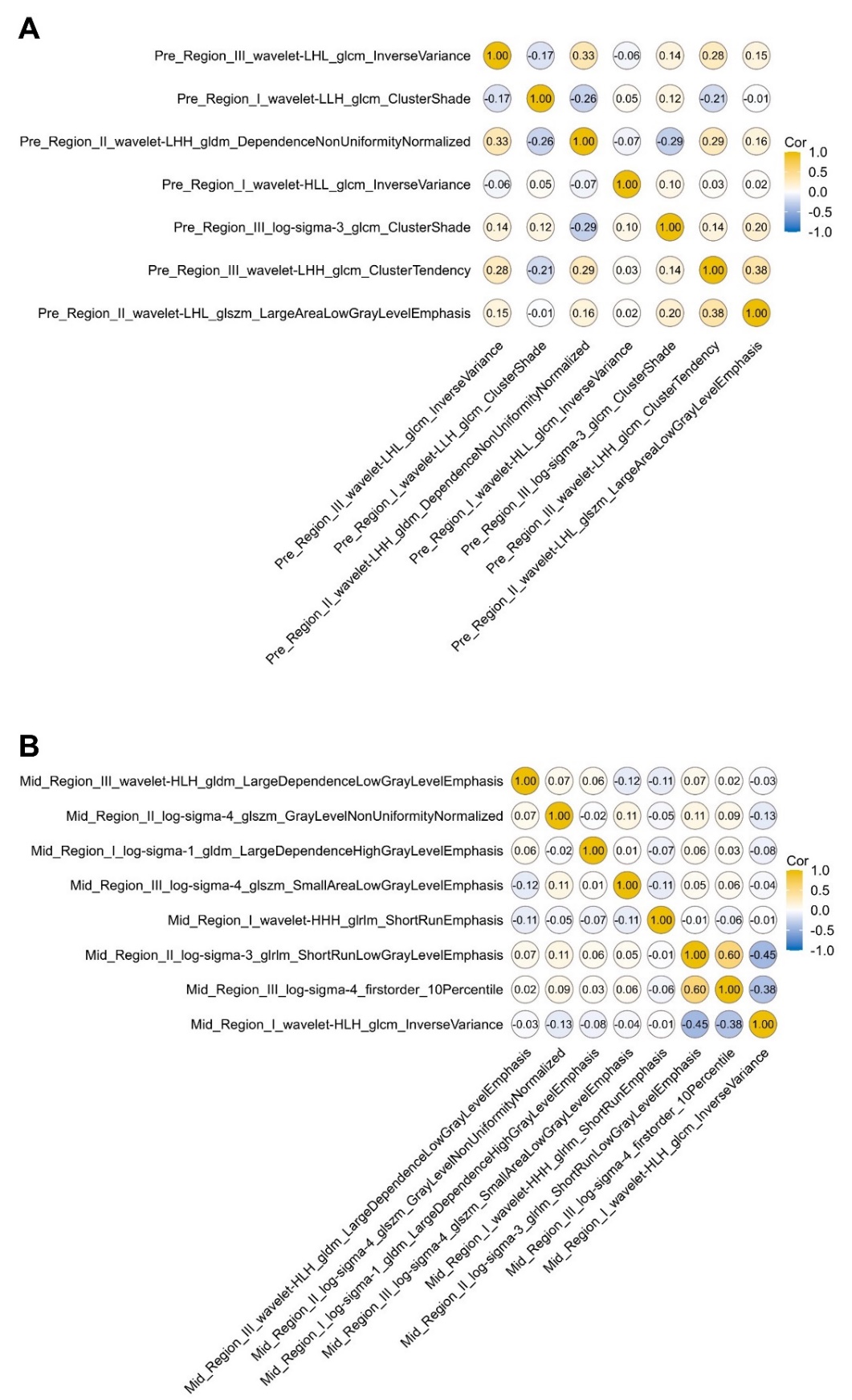


**Fig S3.** The spearman correlation coefficient among habitat radiomics features from (A) pre-NAT and (B) mid-NAT MRI image for predicting pathological complete response.


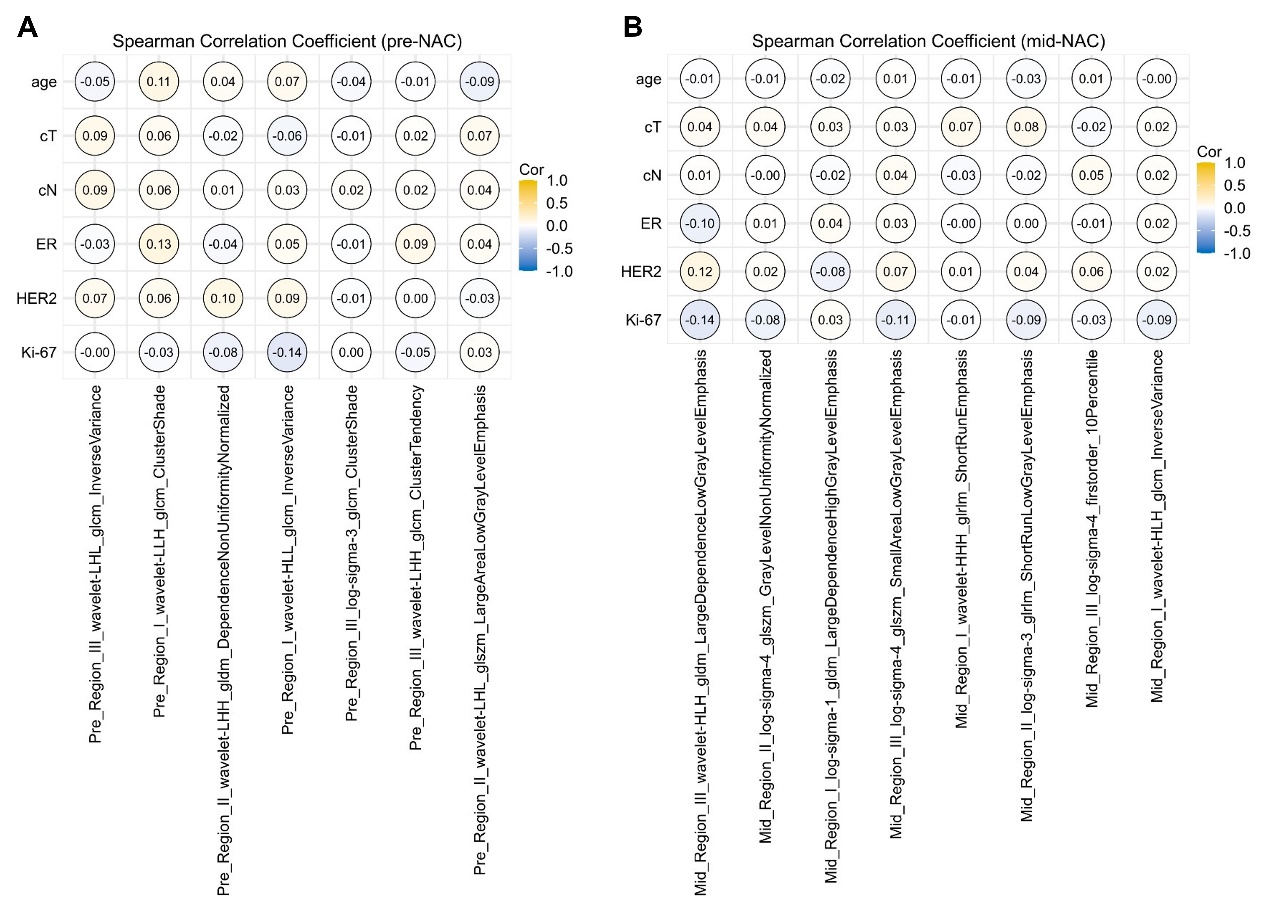


**Fig S4.** the spearman correlation coefficient among radiomics features and clinical characteristics from (A) pre-NAT and (B) mid-NAT MRI images for predicting pathological complete response.


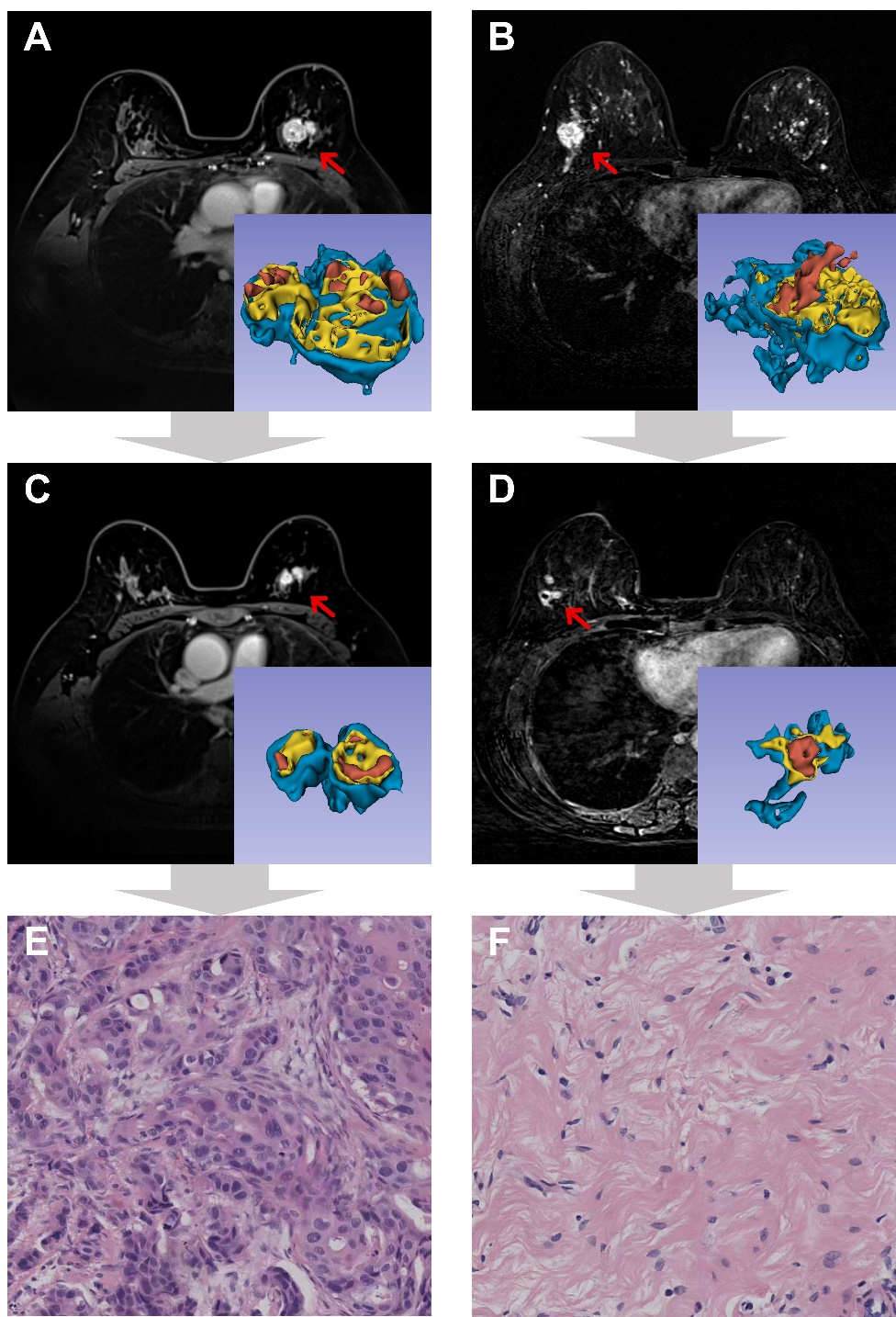


**Fig S5.** The examples of dynamic contrast enhancement T1-weighted MR images, 3D view of entire tumor and spatial habitat, and 40x H&E pathology image of specimen after neoadjuvant chemotherapy in two patients with breast cancer. pre-NAT (A) and mid-NAT (C) MRI images in a 65-year-old woman with HR+/HER2- breast cancer. pre-NAT (B) and mid-NAT (D) MRI images in a 46-year-old woman with HER2+ breast cancer. (E) pathological assessment showed residual invasive cancer after NAT. (F) pathological assessment showed no residual invasive cancer after NAT.


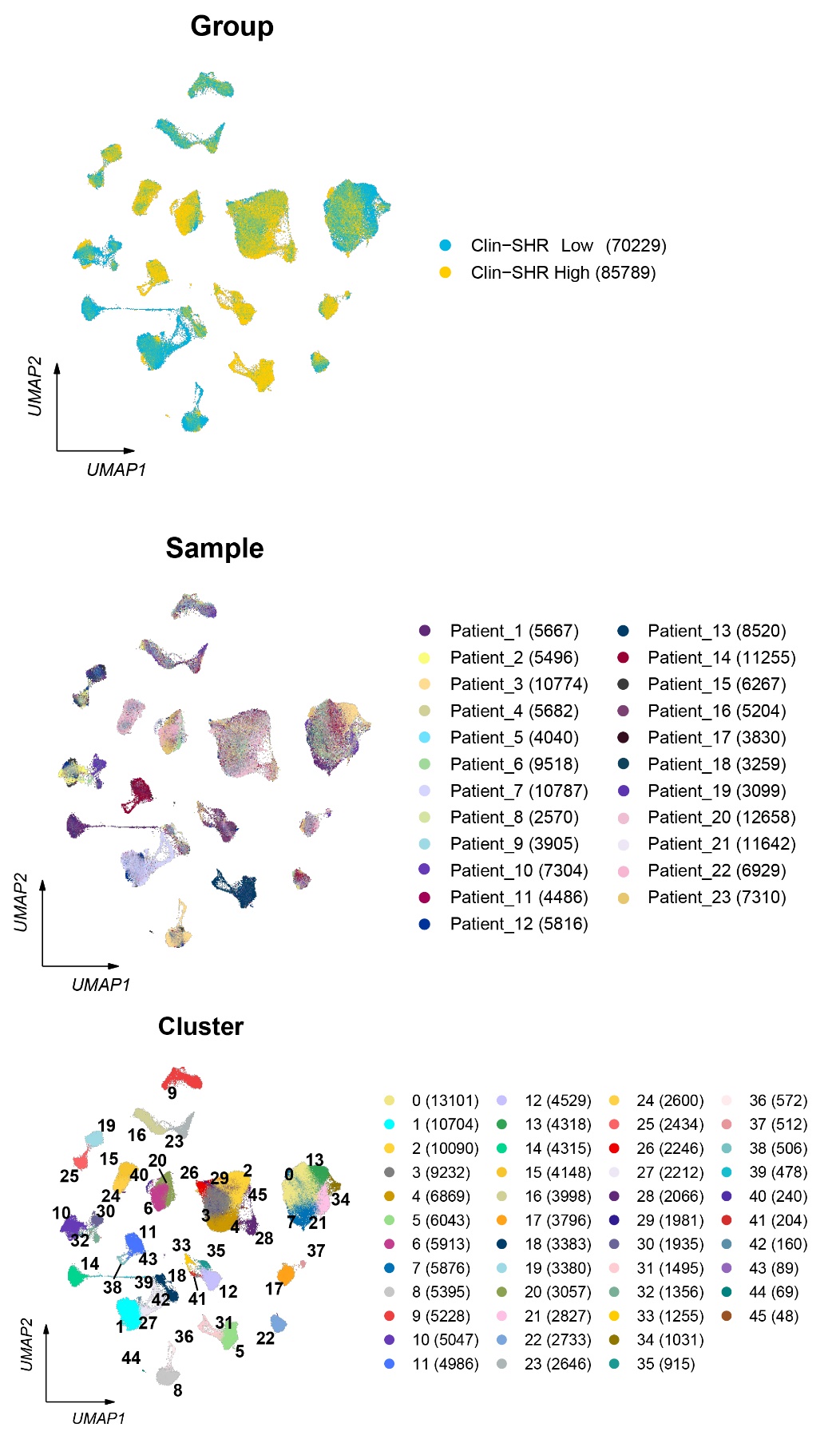


**Fig S6.** The Uniform Manifold Approximation and Projection (UMAP) visualization of single-cell RNA samples in different groups and individual patients. Panel group shows a UMAP projection of cells from Clin-SHR high and low groups. Panel Sample displays the distribution of individual patients' cells. Panel Cluster illustrates the clustering of cells into 46 distinct groups, emphasizing the diversity of the tumor immune microenvironment.





**Fig S7.** The UMAP visualization of cell composition across individual patients. This figure presents UMAP plots of immune cell populations for 23 individual patients (HER2+: 9 patients; and TN: 14 patients), and each plot shows the distribution of various immune cell types.


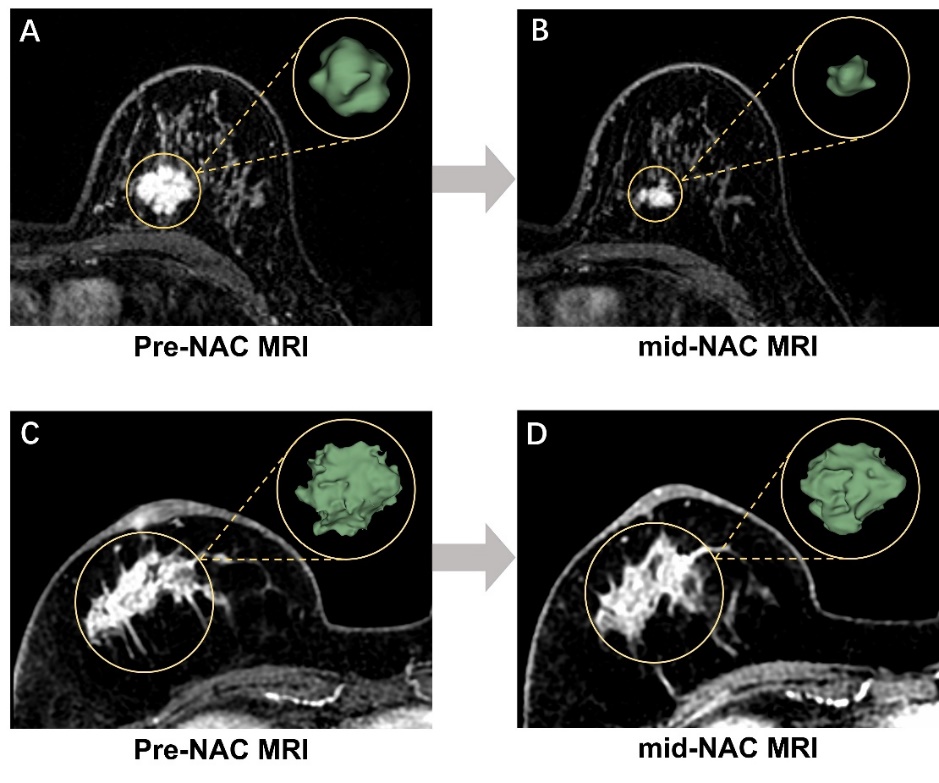


**Fig S8.** The segmentation of tumor region at baseline DCE MRI image, and we delineated the entire tumor region for further analysis. During ROI segmentation, the necrosis, air and calcification area of the breast cancer were including as well. Figure (A) and (B) was from a patient (66 years old; HER2+ breast cancer; cT2 stage) who achieved pathological complete response after neoadjuvant chemotherapy, while Figure (C) and (D) was from a patient (48 years old; TN breast cancer; cT3 stage) who did not achieve pathological complete response after neoadjuvant chemotherapy.
